# SIRT1 regulates the phosphorylation and degradation of P27 by deacetylating CDK2 to promote T-cell acute lymphoblastic leukemia progression

**DOI:** 10.1186/s13046-021-02071-w

**Published:** 2021-08-18

**Authors:** Fangce Wang, Zheng Li, Jie Zhou, Guangming Wang, Wenjun Zhang, Jun Xu, Aibin Liang

**Affiliations:** 1 Department of Hematology, Tongji Hospital, Tongji University School of Medicine, 1239 Siping Road, Shanghai, 200092 People’s Republic of China; 2 East Hospital, Tongji University School of Medicine, 1239 Siping Road, Shanghai, 200092 People’s Republic of China

**Keywords:** T-cell lymphoblastic leukemia, Cell cycle, SIRT1, p27, Phosphorylation, Ubiquitination

## Abstract

**Background:**

Despite marked advances in the clinical therapies, clinical outcome of most T-cell acute lymphoblastic leukemia (T-ALL) patients remains poor, due to the high risk of relapse, even after complete remission. Previous studies suggest that the NAD-dependent deacetylase sirtuin 1 (SIRT1) has a dual role in hematologic malignancies, acting as a tumor suppressor or tumor promoter depending on the tumor type. However, little is known about the expression and functions of SIRT1 in T-ALL leukemogenesis.

**Methods:**

Public RNA-seq data, a Notch1 driven T-ALL mouse model and γ-secretase inhibitor were used to identify SIRT1 expression in T-ALL. We knocked down SIRT1 expression with ShRNAs and assessed the impacts of SIRT1 deficiency on cell proliferation, colony formation, the cell cycle and apoptosis. Transgenic SIRT1 knockout mice were used to determine the function of SIRT1 in vivo. RT-PCR, western blot, co-immunoprecipitation and ubiquitination analyses were used to detect SIRT1, p27 and CDK2 expression and their interactions.

**Results:**

SIRT1 protein expression was positively correlated with the activation of Notch1. Downregulation of SIRT1 expression suppressed the proliferation and colony formation of T-ALL cell lines, which was reversed by SIRT1 overexpression. SIRT1 silencing prolonged the lifespan of T-ALL model mice. We demonstrated that p27 was involved in the downstream mechanism of cell cycle arrest induced by silencing SIRT1. SIRT1 increased the phosphorylation of p27 on Thr187 by deacetylating CDK2 and enhanced the interaction between p27 and SKP2 leading to the degradation of p27.

**Conclusion:**

Our findings suggest that SIRT1 is a promising target in T-ALL and offer a mechanistic link between the upregulation of SIRT1 and downregulation of p27.

**Supplementary Information:**

The online version contains supplementary material available at 10.1186/s13046-021-02071-w.

## Background

T-cell acute lymphoblastic leukemia (T-ALL) is a lethal hematological malignancy notable for its aggressive, metastatic, and chemo-resistant propensities. T-ALL accounts for 10–15% of pediatric ALL cases and 20–25% of ALL cases diagnosed in adults [15, 27]. Although improvements in the efficacy of multiagent chemotherapy have increased the first remission rate to 90%, the prognosis for relapsed T-ALL is dismal with cure rates less than 30% [15]. To address this clinical challenge, recent studies investigating the molecular understanding of T-ALL have paved the way for the development of precision medicine approaches for T-ALL therapy. Genome-wide sequencing identified several genetic mutations or alterations in T-ALL, with Notch1 mutations identified in more than 50% of T-ALL cases [42]. These findings stressed the importance of the Notch pathway in T-ALL.

The NAD-dependent deacetylase sirtuin 1 (SIRT1) is a well-studied deacetylase that deacetylates histones and non-histone proteins. SIRT1 is known to be involved in hematologic malignancies but its role is controversial [5]. SIRT1 is reported to be overexpressed in chronic myeloid leukemia (CML) leukemic stem cells (LSCs) and contributes to LSC maintenance [1, 24]. Another study found that SIRT1 is overexpressed in FLT3-ITD acute myeloid leukemia (AML) LSCs and protects these cells from apoptosis through regulation of p53 acetylation [23]. However, SIRT1 can function as a tumor suppressor in the context of the MLL mutant molecular subtype of AML via deacetylation of local H3K9 histones [6]. Moreover, SIRT1 levels are decreased in myelodysplastic (MDS) hematopoietic stem/progenitor cells (HSPCs), and SIRT1 deficiency in MDS HSPCs enhances their growth and self-renewal [40]. Relatively little is known about the effect of SIRT1 on T-ALL. Heshmati M et al. reported that ghrelin induces the proliferation of T-ALL cells via activation of the SIRT1/AMPK axis, whereas Okasha S M et al. reported that SIRT1 activation suppressed the growth of T-ALL cells by inhibiting Notch, NF-ĸB, and mTOR signaling [13, 33]. Liang L reported that silencing SIRT1 resulted in enhanced apoptosis and cell cycle arrest after etoposide treatment [25].

Previous studies evaluating the role of SIRT1 in T-ALL have been limited by the lack of in vivo models of SIRT1 deficiency. In this study, we used a genetic SIRT1-knockout (KO) model to define the role of SIRT1 in regulating T-cell leukemogenesis. We evaluated the association between Notch1 and increased SIRT1 protein levels, as well as the contribution of SIRT1 to T-ALL cell growth in vitro and leukemogenesis in vivo. SIRT1 inactivation induced cell cycle arrest and accumulation of the cyclin-dependent kinase inhibitor p27. Finally, we investigated the underlying mechanisms connecting SIRT1 and the ubiquitination of p27.

## Methods and materials

### Public RNA-seq data

RNA-seq data of 264 T-ALL, 267 B-ALL and 337 normal samples were downloaded from the ALL Phase 2 project of the TARGET database (https://ocg.cancer.gov/) and the TCGA TARGET GTEx cohort of UCSC Xena project (http://xena.ucsc.edu/). Box plot was drawn with R (http://www.r-project.org/).

### Cells and drug

Cell lines (CCRF-CEM, MOLT4, KG-1, THP-1. MV4–11, K562, U937 and 293 T) were purchased from the Type Culture Collection of the Chinese Academy of Sciences, Shanghai, China. The leukemia cell lines MOLT-4, CCRF-CEM, KG-1, THP-1. MV4–11, U937 were maintained in RPMI 1640 medium (Gibco, Cat No. 61870036) supplemented with 10% fetal bovine serum (FBS, Gibco, Cat No. 12483020) and 1% penicillin/streptomycin (Gibco, Cat No. 15140122). K562 cells were maintained in IMDM medium (Gibco, Cat No. 12440046) containing 10% FBS and 1% penicillin/streptomycin. The 293 T cell was maintained in DMEM medium (Gibco, Cat No. 10566016) containing 10% FBS and 1% penicillin/streptomycin. All cell lines were regularly tested for mycoplasma contamination. DAPT dissolved in DMSO was purchased from Selleck (Cat No. S2215). Cells were treated with a final concentration of 10 μM DAPT for 72 h, then washed and refed medium containing DAPT (mock) or medium lacking DAPT (wash) for 24 h. All human samples were conducted with approval from the ethical review committees of biomedical research of Shanghai Tongji Hospital. T-ALL patient sample information is in Supplementary Table 1.

### DNA constructs

MSCV-IRES-GFP, MSCV-ICN1-IRES-GFP and MSCV-ShRNA-ICN1-IRES-GFP were kindly provided by Professor Hudan Liu, Medical Research Institute, Wuhan University, Wuhan, China. pLVX-ShRNA1, pLVX-ShRNA2 and pLVX-IRES-ZsGreen vectors were purchased from Clontech Laboratories. Hemagglutinin (HA)-Ubiquitin, pMD2.G, psPAX2 and pCL-Eco were purchased from Addgene. pCMV-Blank, pCMV-N-MycTag and pCMV-N-Flag were purchased from Beyotime Biotechnology. A modified pLVX-ShRNA2-mCherry vector in which the ZsGreen fluorescent marker was replaced by an mCherry fluorescent marker was made using HiFi DNA Assembly Master Mix (NEB, Cat No. E2621L). ShSIRT1 and ShScramble were cloned into pLVX-ShRNA2-mCherry. ShScramble, ShMYC and Shp27-human were cloned into pLVX-ShRNA2. The SIRT1 coding region was cloned into pCMV-N-MycTag, pCMV-N-Flag and pLVX-IRES-ZsGreen. Mutant SIRT1-H363Y vector was generated using Q5® site-directed mutagenesis kit (NEB, Cat No. E0554). Sanger sequencing of the SIRT1-H363Y plasmid verified the presence of the desired mutation (Fig. S3a). CDK2, SKP2, MYC and mCherry were cloned into pCMV-Blank. CDK2 and p27 were cloned into pCMV-N-Flag. Shp27-mouse and ShRen were cloned into MSCV-ShRNA-ICN1-IRES-GFP.

### ShRNA sequences

The anti-SIRT1 ShRNA sequences used in this study were named ShSIRT1. A scrambled sequence was used as a control for off target effects of ShRNA. The sequences were as follows: ShSIRT1–1: TGGCCATTTCCCTACTTATAA, ShSIRT1–2: GAAGTGCCTCAGATATTAA, ShScramble: GCGCGCTTTGTAGGATTCG, ShMYC-1: CAGTTGAAACACAAACTTGAA, ShMYC-2: CCTGAGACAGATCAGCAACAA, Shp27-human: AGCAATGCGCAGGAATAAGG, Shp27-mouse: CGCAAGTGGAATTTCGACTTT and ShRen: AGGAATTATAATGCTTATCT.

### Mice and animal procedures

All animals were housed in specific pathogen-free facilities at the animal experiment center of Tongji University. All the animal experiments were performed in accordance with institutional guidelines for animal care at Tongji University School of Medicine and received ethical approval from the Animal Ethics Committee of Tongji University.

SIRT1^co/co^ mice (Cat No. 008041) and Mx1-Cre transgenic mice (Cat No. 003556) were purchased from the Jackson Laboratory [21, 22]. Sexually mature SIRT1^co/co^ and Mx1-Cre mice were crossed to obtain Mx1-Cre SIRT1^+/+^ or Mx1-Cre SIRT1^co/co^ mice. For inducible ablation of SIRT1, 6 weeks old Mx1-Cre SIRT1^co/co^ and Mx1-Cre SIRT1^+/+^ mice were intraperitoneally injected with poly I:C (10 mg/kg, Sigma-Aldrich, Cat No. P1530) every other day for a total of five times. The mice were sacrificed 4 days after the last injection for collection of HSPCs. Genotyping analysis of tail DNA was performed utilizing Mouse SIRT1-co-gDNA primer pair. Genotyping analysis of bone marrow cDNA was performed using a forward primer located in the exon 3 and a reverse primer located in exon 5 (Mouse SIRT1-ko-cDNA primer pair).

Female C57BL/6 J mice (8 weeks old) were purchased from Shanghai SLAC Laboratory Animal Company and used as recipients in all mouse leukemia-model experiments.

B6.SJL (CD45.1) mice were provided by Professor Caiwen Duan at Shanghai Jiao Tong University School of Medicine (Shanghai, China) and were used as transplant recipients in a homing assay.

### Soft agar assays

Cells were plated in 24-well plates in a two-layer soft agar system (0.6% agarose on the bottom and 0.3% agarose on the top) with 200 cells per well in a volume of 400 μl per well as previously described [32].

### Immunoblotting

Cells were lysed with RIPA lysis and extraction buffer (Thermo Scientific, Cat No. 89901) with the phosphatase inhibitor cocktail (EpiZyme, Cat No. GRF102) and the protease inhibitor cocktail (EpiZyme, Cat No. GRF101) and boiled in 5X SDS-PAGE sample loading Buffer (Beyotime, Cat No. P0015). Total cellular proteins were resolved by SDS-PAGE, transferred to nitrocellulose membranes (Amersham, Cat No. 10600001), blocked with 5% fat free milk in TBS/0.05% Tween-20 and incubated with the indicated primary antibody overnight at 4 °C. Then, the membranes were probed with appropriate horseradish peroxidase-conjugated secondary antibodies for 1 h at room temperature. The protein bands were visualized with an enhanced chemiluminescent substrate (NCM biotech, Cat No. P10200) on Imager 600 machine (Amersham). For cycloheximide (CHX) assay, cells were treated with 100 μM CHX (Sigma, Cat No. 5087390001) for 2, 4 or 8 h prior to lysis. Antibodies used for immunoblotting are listed in Supplementary Table 2.

### RNA extraction and quantitative real-time PCR

Total cellular RNA was extracted using TRIzol (Invitrogen, Cat No. 15596026) and reverse transcribed using a PrimeScript RT reagent kit (Takara, Cat No. RR037A) according to the manufacturer’s instructions. Quantitative PCR (Q-PCR) was performed using SYBR Premix Ex Taq II (Takara, Cat No. RR820A) on a LightCycler 96 PCR system (Roche Diagnostics). Relative mRNA expression was calculated by the comparative Ct method and the results were normalized to the glyceraldehyde-3-phosphate dehydrogenase (GAPDH) results for each sample. The sequences of the PCR primers used are listed in Supplementary Table 3.

### Lentiviral or retroviral transduction

For lentiviral vector production, pLVX-ShRNA1, pLVX-ShRNA2, pLVX-IRES- ZsGreen and pLVX-IRES-mCherry vectors were used for plasmid construction and transfected into 293 T cells with packaging plasmids (pMD2.G and psPAX2). For retroviral vector production, retroviral vectors MSCV-IRES-GFP, MSCV-ICN1-IRES-GFP and a modified MSCV-IRES-GFP vector were used for plasmid construction and transfected into 293 T cells with a helper plasmid (pCL-Eco) [39]. Viral supernatants were generally harvested 2 days after transfection. T-ALL cells were infected by spinoculation (1000 g for 2 h at 32 °C, 4 μg/μl polybrene). After spinoculation, the cells were then supplemented with 3 ml fresh medium and cultured for an additional 48 h.

### Immunofluorescence assay

Cells were fixed with 4% paraformaldehyde, permeabilized with 0.2% Triton X-100 in PBS for 5 min and blocked with 3% bovine serum albumin (BSA) for 1 h. Cells were incubated with an anti-FLAG tag (D6W5B) rabbit monoclonal antibody (mAb) (Cell Signaling Technology, Cat No. #14793, 1:100) and anti-Myc tag (9B11) mouse mAb (Cell Signaling Technology, Cat No. #2276, 1:100) at 4 °C overnight, followed by treatment with a goat anti-rabbit IgG secondary antibody conjugated to Alex Fluor 488 (Invitrogen, Cat No. R37116, 1:200) and donkey anti-mouse IgG secondary antibody conjugated to cyanine cy5 (Jackson ImmunoResearch, Cat No. 715–175-150, 1:200).

### Establishment of Notch1-induced leukemia model

To establish Notch1-induced T-ALL leukemia in mice, donor mice (weeks 6–8) were sacrificed and lineage-negative HSPCs were isolated with lineage cell depletion kit (Miltenyi Biotec, Cat No. 130–090-858). Retroviruses expressing MSCV-IRES-GFP (empty vector), MSCV-ICN1-IRES-GFP, or MSCV-Shp27-ICN1-IRES-GFP were transduced into HSPCs by spinoculation (1000 g for 2 h at 32 °C; 4 μg/μl polybrene). The cells were then cultured with RPMI 1640 medium supplemented with 20% FBS, 1% penicillin/streptomycin, IL-3 (PeproTech, Cat No. 213–13, 10 ng/ml), IL-6 (PeproTech, Cat No. 216–16, 10 ng/ml), and SCF (PeproTech, Cat No. 250–03, 100 ng/ml) overnight. A second round of spinoculation was performed 24 h later. After washing with PBS, cells were injected intravenously into lethally irradiated (9 Gy) recipients (1 × 10^6^ cells per mouse). The mice were maintained on antibiotic-treated water. The mice were bled every week to monitor blood counts and evaluate the percentage of GFP^+^ cells by flow cytometry. For the 2nd transplantation, a specific number of GFP^+^ cells sorted from the bone marrow of 1st transplantation recipient mice were injected intravenously into lethally irradiated (9 Gy) recipients together with 1 × 10^6^ unfractionated bone marrow cells for hemogenic support.

### Homing assay

For examining homing ability of SIRT1 knock-out (KO) and wild-type (WT) bone marrow (BM) cells, a total of 5 × 10^6^ BM cells from KO and WT mice were transplanted into lethally irradiated recipients (CD45.1). 24 h after transplantation, donor-derived cells (CD45.2) in the BM were detected by FACS. For examining homing ability of SIRT1 KO and WT T-ALL cells, a total of 1 × 10^6^ leukemia cells (GFP^+^) from KO and WT T-ALL were transplanted into lethally irradiated recipients. 24 h after transplantation, GFP^+^ cells in the BM were detected by FACS.

### Flow cytometric analysis for the detection of apoptosis and the cell cycle distribution

To detect apoptosis, cells were washed with PBS and resuspended with Annexin V binding buffer at 1 × 10^6^ cells/ml. 100ul cell suspension was stained with 5 ul APC-labeled Annexin V (BioLegend, Cat No. 640932) at room temperature for 1 h, and 10ul propidium iodide (PI) solution was added 10 min before flow cytometric analysis. Bromodeoxyuridine (BrdU, MedChemExpress, Cat No. HY-D0184) and PI staining was performed to detect the cell cycle distribution. Cells (1 × 10^6^) were plated in 100 mm dishes with 10 μM BrdU for 1 h before harvesting. For detection of the cell cycle distribution in the T-ALL model, KO and WT mice received an initial intraperitoneal injection of BrdU (1 mg/6 g mouse weight) and were then maintained on 1.0 mg/ml BrdU in the drinking water for 24 h prior to sacrifice. Cells treated with BrdU were fixed in 75% ethanol at − 20 °C for at least 2 h and processed according to manufacturer’s instructions. An APC-labeled anti-BrdU antibody (BioLegend, Cat No. 364114) was used to detect BrdU, and a PI solution was added 10 min before flow cytometric analysis.

### Co-immunoprecipitation assays

Cells for co-immunoprecipitation assay were lysed in Pierce IP lysis buffer (Thermo Scientific, Cat No. 87787) containing a protease inhibitor cocktail. For immunoprecipitation of FLAG or MycTag, the whole cell lysates were incubated with anti-Flag magnetic beads (Bimake, Cat No. B26101), anti-Myc magnetic beads (Bimake, Cat No. B26301) or Mouse IgG Magnetic beads (Cell Signaling Technology, Cat No. #5873) respectively overnight at 4 °C with rotation. After washing three times with PBST buffer, the immune complexes were boiled in SDS sample loading buffer and subjected to western blot analysis.

### Ubiquitination assay

293 T cells were transduced with vectors as indicated with HA-tagged ubiquitin constructs for 48 h. Before harvesting, cells were treated with 10 μM MG132 for 24 h (MedChemExpress, Cat No. HY-13259). The cells were then lysed in Pierce IP lysis buffer containing a protease inhibitor cocktail and immunoprecipitated with anti-Flag magnetic beads. Ubiquitination was detected by using an anti-HA (F-7) antibody (Santa Cruz, Cat No. sc-7392).

### Pull-down assay

SIRT1-GST, SIRT1-HIS, CDK2-GST, CDK2-HIS fusion protein was expressed in E coli. and purified using the GST-tagged fusion protein purification kit and BeyoGold™ His-tag Purification Resin and BeyoGold™ GST-tag Purification Resin according to the manufacturer’s instructions. Recombinant GST fusion proteins were incubated with HIS-tagged protein in vitro. The protein complexes were then pulled down with GST beads, eluted with SDS sample buffer, and resolved by SDS/PAGE.

### Statistics

Data obtained from independent experiments are reported as the mean ± SEM. Log-rank analysis was used for comparison of differences in Kaplan–Meier survival curves. The unpaired Student’s t test, Mann-Whitney test, and two-way ANOVA with multiple comparisons were applied as appropriate. *p* < 0.05 was considered statistically significant.

## Results

### SIRT1 is highly expressed in T-ALL

**Fig. 1:**
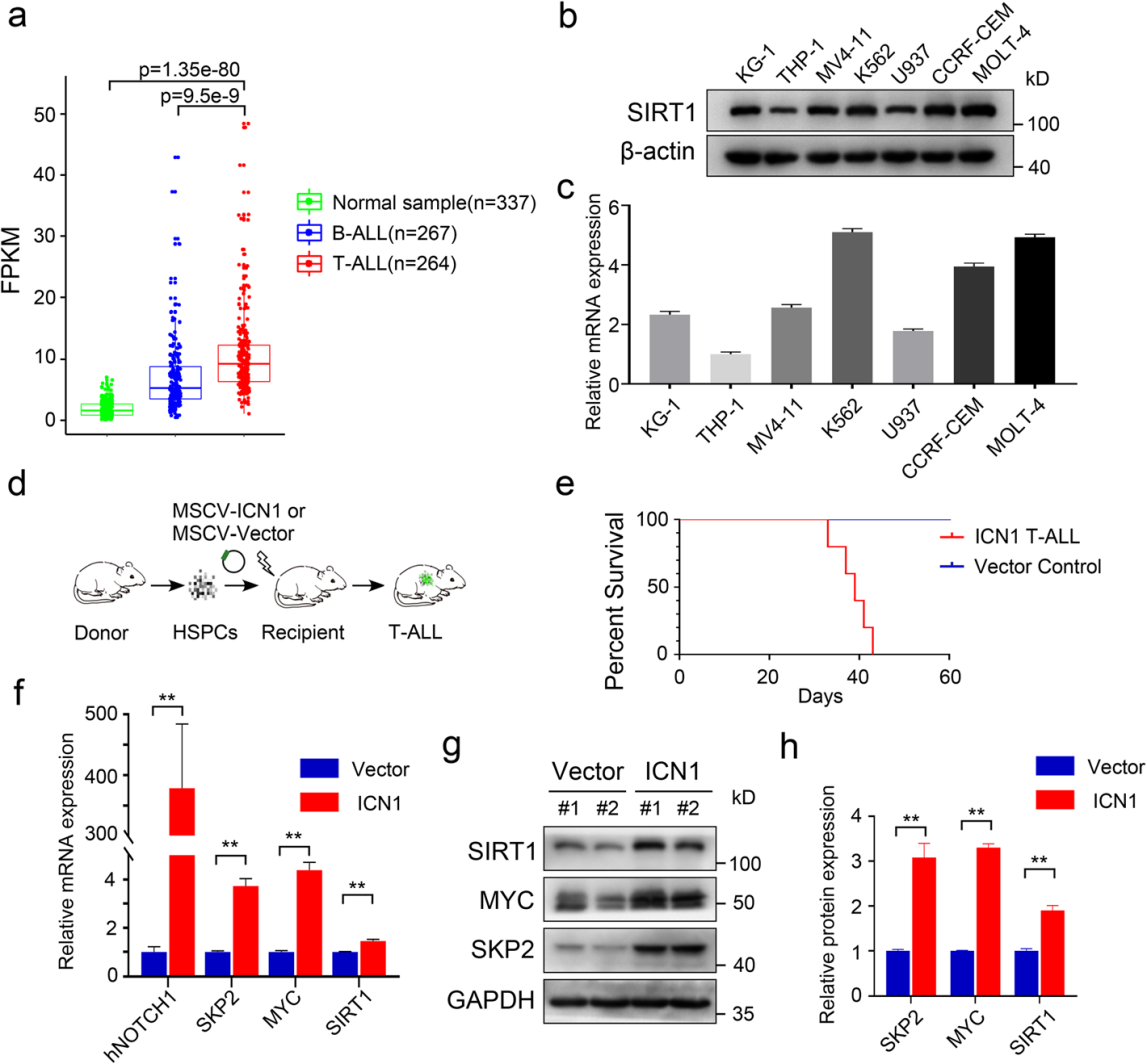
High SIRT1 expression in T-ALL. **a** RNA-seq expression profiles of SIRT1 in the T-ALL, B-ALL and normal samples from public database. **b** Immunoblots of SIRT1 in leukemia cell lines. **c** SIRT1 mRNA expression in leukemia cell lines. **d** Schematic representation of murine T-ALL model. **e** Kaplan-Meier survival curves of mice harboring MSCV-ICN1 and MSCV-Vector HSPCs (*n* = 5 per group). **f** SIRT1 mRNA expression in T-ALL mouse model and control group. **g-h** Immunoblots and relative quantification expression of SIRT1 in T-ALL mouse model and control group (*n* = 3)

We first studied SIRT1 expression in public data base and found that the level of SIRT1 mRNA expression was higher in T-ALL than in normal samples or B-ALL (Fig. 1a). Then we measured the protein levels of SIRT1 in different types of human cell lines by western blot and Q-PCR analysis. SIRT1 was highly expressed at both protein and mRNA levels in T-ALL cell lines MOLT-4 and CCRF-CEM (Fig. 1b-c). We then transduced murine HSPCs with the intracellular form of Notch1 (MSCV-ICN1-IRES-GFP) or the Vector control (MSCV-IRES-GFP) and transplanted into lethally irradiated mice (Fig. 1d). Recipients of ICN1 transduced HSPCs showed accumulation of GFP^+^ T cells with CD4^+^ CD8^+^ immunophenotype in peripheral blood (PB), organ infiltration and succumb to leukemia (Fig. 1e, S1a-c). As anticipated, ICN1 overexpression resulted in activation of the expression of the known Notch targets MYC and SKP2 at the mRNA and protein levels. Although the SIRT1 mRNA level was modestly elevated, the SIRT1 protein level was significantly increased in the T-ALL mouse model (Fig. 1f-h). This would suggest that the increased SIRT1 level was also due to a posttranscriptional mechanism.

### SIRT1 protein level was increased by Notch1/Myc axis in T-ALL

**Fig. 2:**
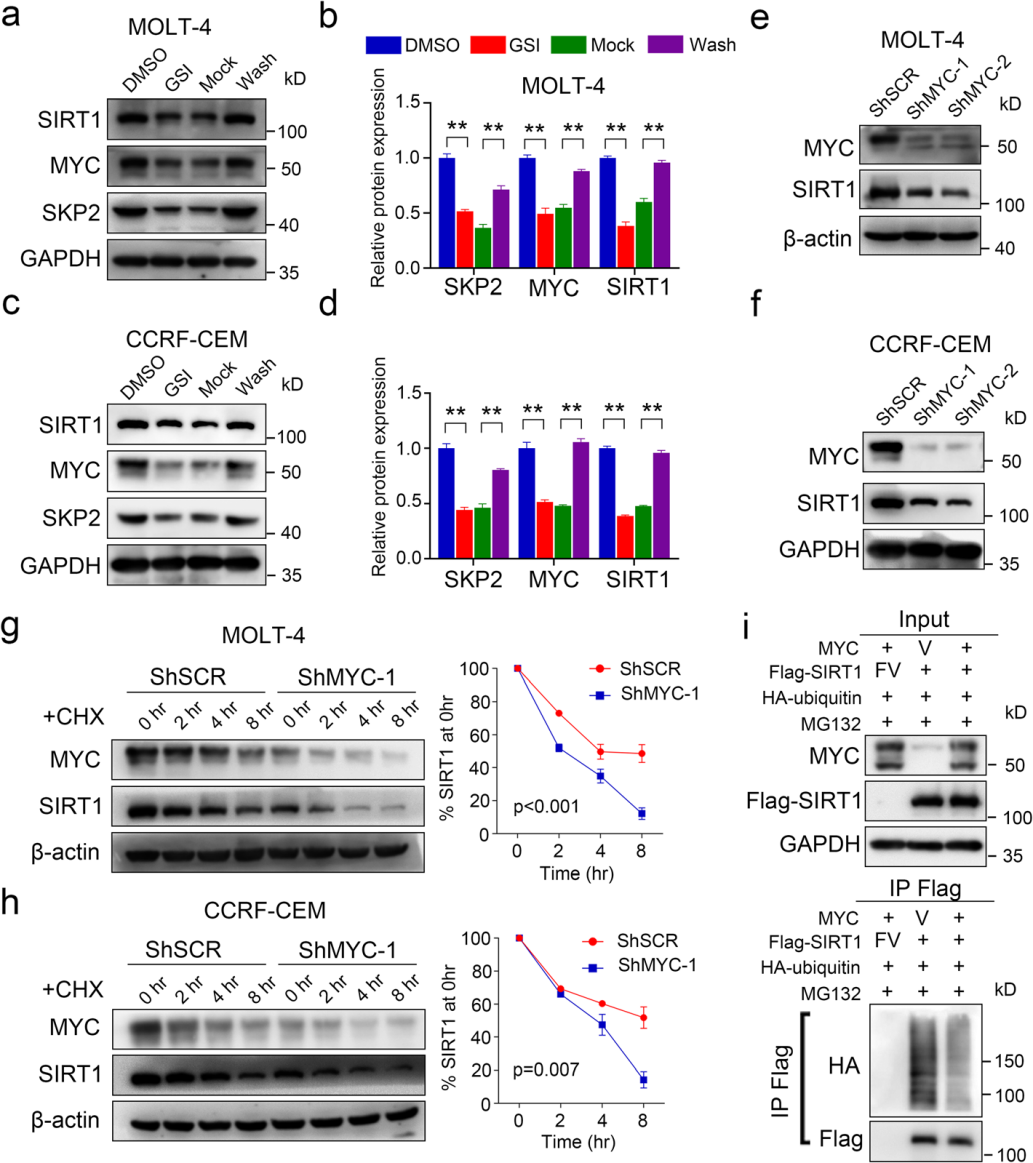
Increased SIRT1 protein levels in T-ALL with Notch1 mutation. **a-d** Immunoblots and relative quantification expression of SIRT1 in MOLT-4 and CCRF-CEM cells treated with GSI, then washed and refed medium containing DAPT (mock) or medium lacking DAPT (wash). **e-f** Western blotting for SIRT1 and MYC levels in MYC-ShRNA-expressing MOLT-4 and CCRF-CEM cells. **g-h** Western blotting for SIRT1 following CHX treatment of MYC knockdown or control MOLT-4 and CCRF-CEM cells (left panel). The right panel showed results of densitometry quantitation (*n* = 3). **i** 293 T cells were co-transfected with pCMV-N-Flag (Flag-Vector, FV), Flag-SIRT1, pCMV-Blank (Vector, V), pCMV-MYC and HA-tagged ubiquitin as indicated and subjected to ubiquitination analysis

A previous study suggested that MOLT-4 and CCRF-CEM cells have mutations within the Notch1 gene [42]. We then observed that the γ-secretase inhibitor (GSI) DAPT blocked Notch signaling induced SIRT1 downregulation at protein level. Removal of DAPT reversed the GSI-mediated inhibition of SIRT1 protein level but had no effect on mRNA level (Fig. 2a-d, S2 a-b). MYC has been proven to be a direct downstream target of Notch1 that contributes to the growth of T-ALL cells [43]. Previous studies have shown an association between MYC and increased SIRT1 protein expression [23, 30]. To evaluate the role of MYC in regulating SIRT1 expression, we inhibited MYC expression using MYC-specific ShRNAs (ShMYC-1 and ShMYC-2). MYC knockdown only reduced SIRT1 protein expression but not mRNA in MOLT-4 and CCRF-CEM cells (Fig. 2e-f, S2 c-d). Inhibition of MYC expression led to accelerated SIRT1 protein decreases in MOLT-4 and CC RF-CEM cells following CHX treatment (Fig. 2g-h). Furthermore, the SIRT1 protein decrease resulting from GSI treatment can be rescued by MYC overexpression, supporting that NOTCH regulates the protein levels of SIRT1 via MYC in T-ALL (Fig. S2e-f). 293 T cells were co-transfected with HA-ubiquitin, Flag-SIRT1, and MYC to evaluate the role of MYC in regulating SIRT1 ubiquitination and the result showed that overexpression of MYC inhibited the polyubiquitination of SIRT1 (Fig. 2i).

### SIRT1 expression promotes the proliferation of T-ALL cells

**Fig. 3:**
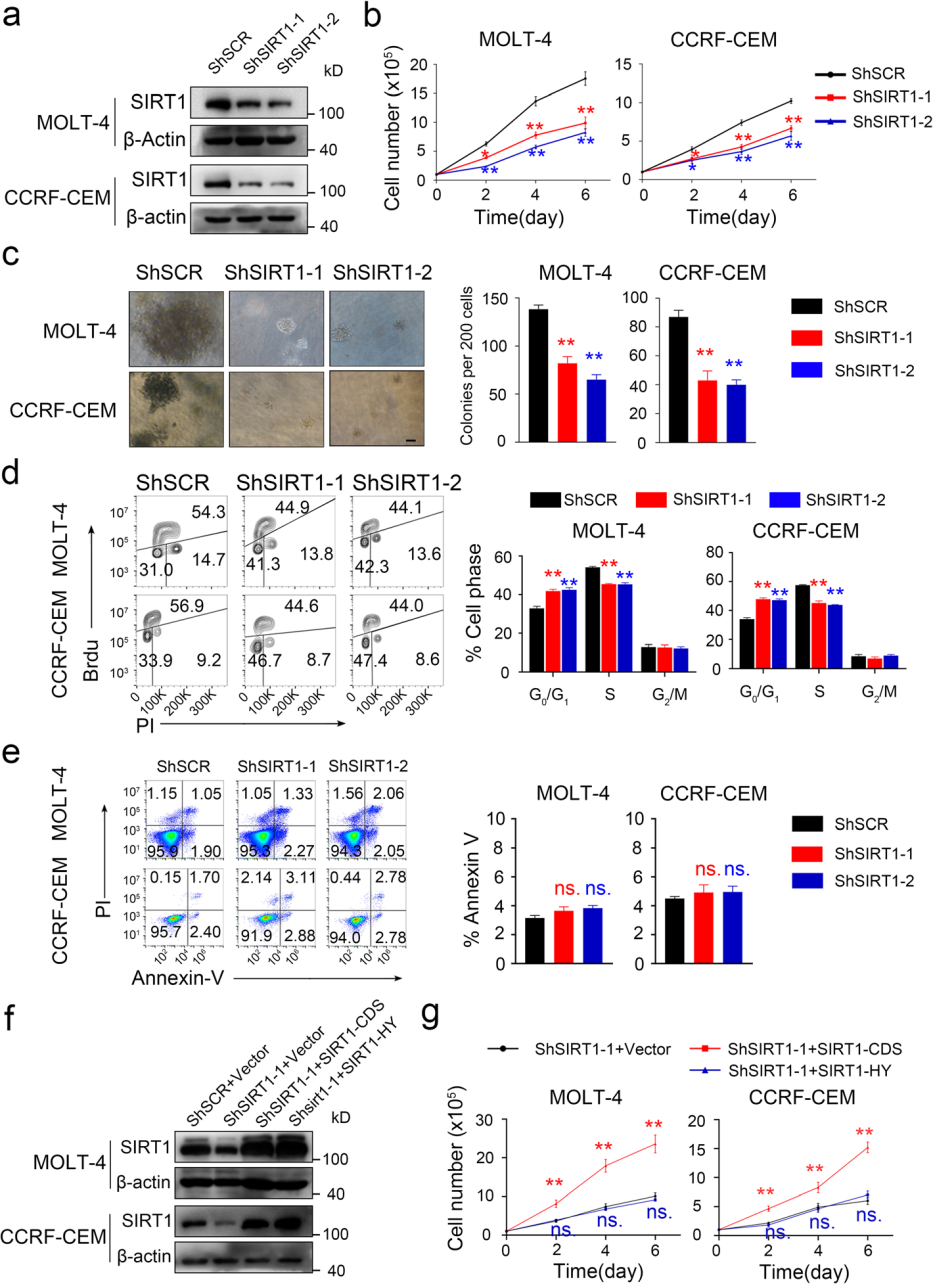
SIRT1 knockdown impairs proliferation. **a** SIRT1 protein levels were analyzed in MOLT-4 and CCRF-CEM cells infected with ShRNA. **b** ShSIRT1 and ShSCR cells were counted and plotted at the indicated time points(n = 3). **c** The left panel showed representative of colonies of ShSIRT1 and ShSCR cells (scale = 200 μm). The right panel showed colony numbers of three independent trials. **d** Cell cycle phase distribution of ShSIRT1 cells were analyzed by FACS and quantified (*n* = 3). **e** Apoptotic cell death was analyzed by Annexin V-PI staining and quantified (n = 3). **f** Western blotting for SIRT1 expression in cells transduced with shSIRT1–1 and empty vector (Vector), coding sequences of SIRT1 (CDS) or SIRT1 deacetylase mutant (SIRT1-HY). **g** Cell growth were counted and plotted at the indicated time points(n = 3)

To investigate the effects of SIRT1 on T-ALL cells, two ShRNAs (ShSIRT1–1 and ShSIRT1–2) were designed to inhibit SIRT1 expression (Fig. 3a). MOLT-4 and CCRF-CEM cells with SIRT1 knockdown proliferated less than their parental counterparts and exhibited growth inhibition in a colony formation assay (Fig. 3b-c). Flow cytometry analysis of BrdU (S-phase) and PI (DNA content) showed arrested cell cycle entry (increased G0/G1-phase distribution, and decreased S-phase distribution) in ShSIRT1 MOLT-4 and CCRF-CEM cells compared to cells transduced with the ShRNA control (Fig. 3d). However, ShSIRT1 did not increase tumor cell apoptosis compared to the scramble control (Fig. 3e). ShSIRT1–1 was designed to target the untranslated region (UTR) region of the SIRT1 mRNA transcript, and therefore, it did not have any effect on a construct containing the coding region of SIRT1. It was reported that SIRT1-H363Y was a deacetylase-inactive point mutant of SIRT1 [8]. To exclude off-target effects of ShRNA, we overexpressed a SIRT1 coding region construct (SIRT1-CDS) or a SIRT1 deacetylase-mutant construct (SIRT1-H363Y) in T-ALL cell lines expressing ShSIRT1–1. The expression of SIRT1-CDS and SIRT1-H363Y maintained SIRT1 protein levels after ShSIRT1–1 transduction (Fig. 3f). Expression of SIRT1-CDS abrogated the ability of ShSIRT1 to inhibit T-ALL cell growth, while expression of SIRT1-H363Y did not produce this effect (Fig. 3g). SIRT1 is nicotinamide adenine dinucleotide (NAD+) dependent and is inhibited by nicotinamide. T-ALL cell viability was reduced by nicotinamide in a dose-dependent manner (Fig. S3b-c).

### Deletion of SIRT1 inhibits oncogenic Notch1-induced transformation of hematopoietic progenitors

**Fig. 4:**
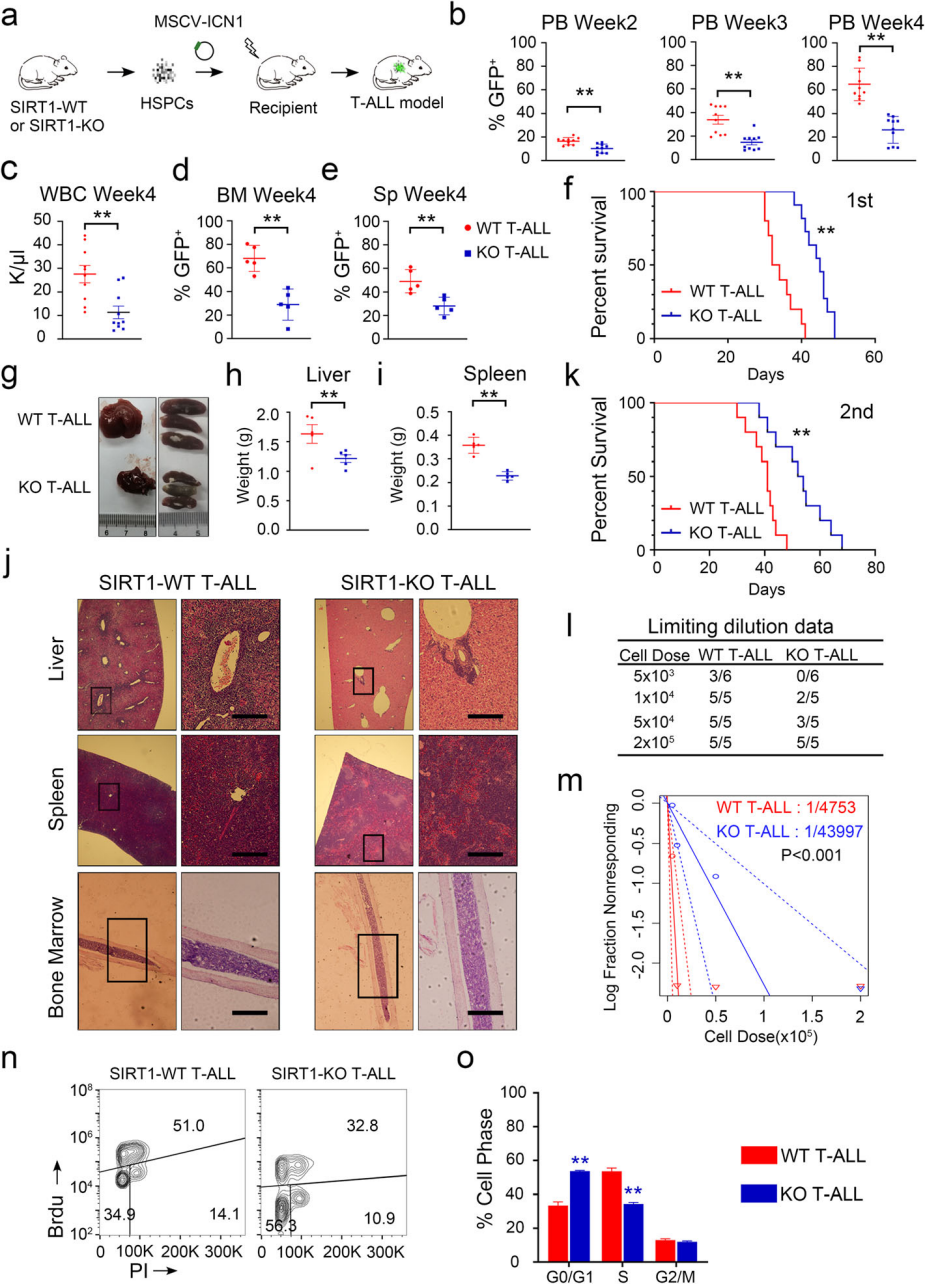
SIRT1 knockout significantly decreases leukemia burden in the murine T-ALL model. **a** Schematic representation of murine T-ALL model. **b** Peripheral blood (PB) percentage of GFP^+^ cells (*n* = 10 per group) at the indicated time points. **c** Cell counts of WBC in the PB of recipient mice at 4 weeks after transplantation. **d-e** Quantification of GFP^+^ cell infiltration in the bone marrow and spleen of mice 4 weeks after transplantation (*n* = 5 per group) **f** Kaplan-Meier survival curves of mice harboring SIRT1 KO and WT T-ALL (n = 10 per group) **g-i** Representative images and quantification of the spleens and livers. **j** Histological analysis of livers, spleens and bones (scale = 5 mm). **k** Kaplan-Meier survival curves of mice upon the second transplantation (cell dose = 1 × 10^6^ GFP^+^ cells per mouse, n = 10 per group). **l-m** Fraction of secondary recipients that developed leukemia when transplanted with limiting dilutions of GFP^+^ cells. **n** Representative images of the cell cycle status in WT and KO T-ALL. **o** Results of cell cycle phase distribution in WT and KO T-ALL (n = 5 per group)

To directly assess the role of SIRT1 loss as a leukemia-initiating event in T-ALL transformation, we crossed a mouse line containing floxed alleles for SIRT1 exon 4 with a Mx1-Cre transgenic mouse line to delete SIRT1 exon 4 (Fig. S4a-d). HSPCs from SIRT1 KO (SIRT1^−/−^ Mx1-Cre) and WT (SIRT1^+/+^ Mx1-Cre) mice were infected with retroviruses driving the expression of ICN1 and transplanted into lethally irradiated mice (Fig. 4a). SIRT1 KO T-ALL recipients exhibited lower GFP^+^ cell percentages and white blood cell counts than SIRT1 WT T-ALL recipients by peripheral blood monitoring (Fig. 4b, c). The frequencies of GFP^+^ cells in the bone marrow and spleen were also lower in the SIRT1 KO T-ALL group (Fig. 4d, e). Knocking out SIRT1 significantly extended the survival of T-ALL mouse model and reduced spleen and liver size (Fig. 4f-i). Similarly, hematoxylin and eosin (HE) staining indicated reduced infiltration of leukemia cells into the liver and spleen (Fig. 4j). Since the extent of leukemia cell maturation often correlates with prognosis, peripheral blood GFP^+^ cells were evaluated for surface markers [44]. Knocking out SIRT1 did not change the CD4^+^ CD8^+^ phenotype of leukemia cells (Fig. S4e). Consistently, SIRT1 KO T-ALL mice prolonged survival during the secondly transplantation (Fig. 4k). To quantify the leukemia- initiating cell (LIC) frequencies in SIRT1 KO or WT T-ALL, equivalent numbers of GFP^+^ cells sorted from primary leukemia model mice were transplanted into secondary recipients at a range of doses from 5 × 10^3^–2 × 10^5^ cells per recipient. Limiting dilution analysis showed that the deletion of SIRT1 resulted in decrease in the LIC frequency compared with the WT counterparts (1 in 43,997 GFP^+^ cells vs. 1 in 4753 GFP^+^ cells, Fig. 4l, m). To rule out the possibility that a failure of cell homing caused the reduction of T-ALL, we injected an equal number of SIRT1 WT or SIRT1 KO BM cells into lethally irradiated CD45.1 mice. FACS analysis showed that the percentages of SIRT1 WT and SIRT1 KO donor-derived cells identified by CD45.2 expression were similar 24 h after bone marrow transplantation (Fig. S4f). We transplanted equal numbers of SIRT1 WT T-ALL and SIRT1 KO T-ALL into recipients and the percentages of GFP^+^ cells in the bone marrow of SIRT1 WT T-ALL and SIRT1 KO T-ALL recipients were similar after 24 h (Fig. S2g). Consistently, BrdU-PI FACS analysis showed that SIRT1 loss blocked cell cycle entry (Fig. 4n, o).

### SIRT1 decreased p27 protein levels in T-ALL cell lines and mouse model

**Fig. 5:**
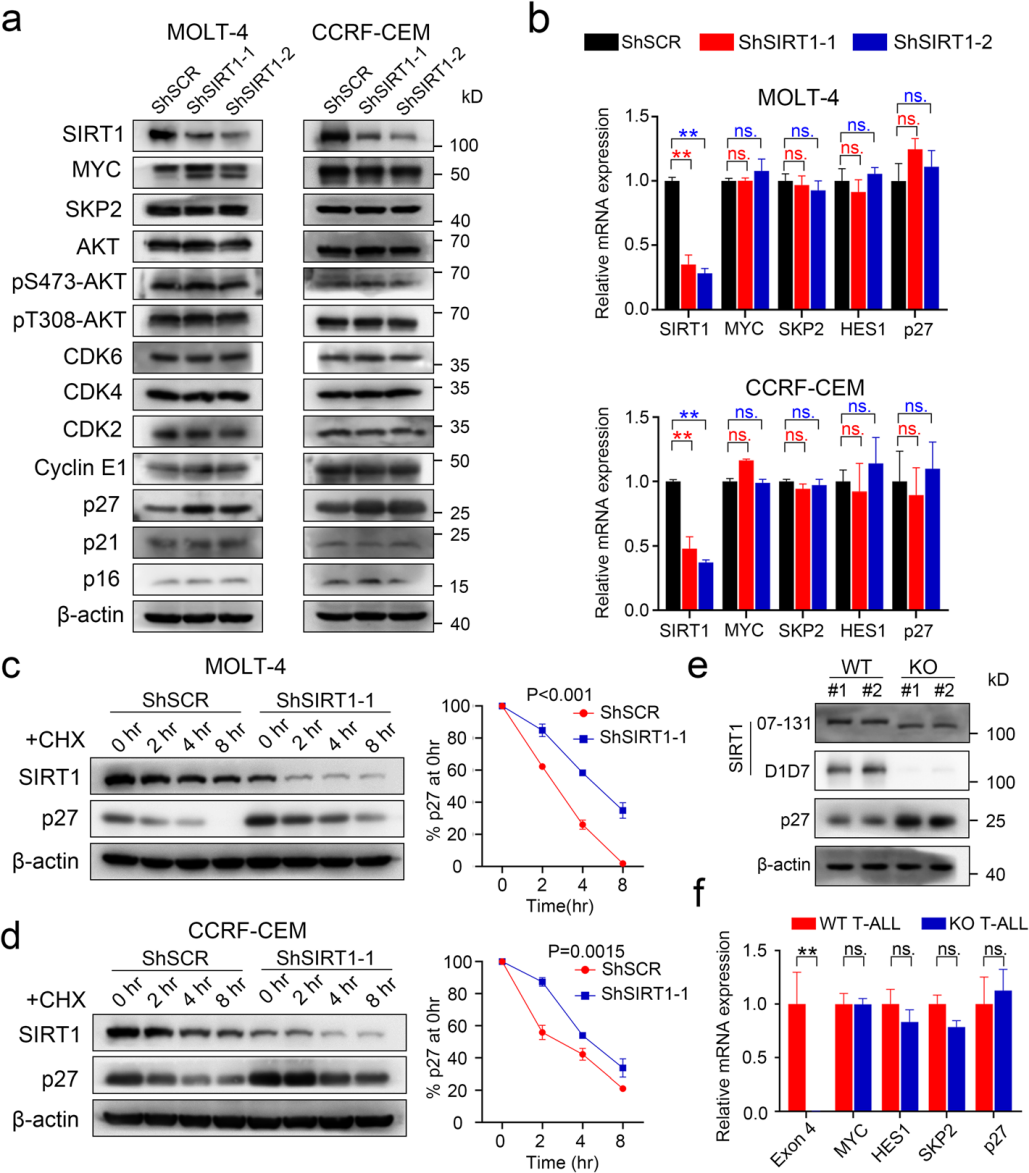
SIRT1 decreases p27 protein levels. **a** Western blot analysis of proteins associated with cell cycle entry in SIRT1 knockdown MOLT-4 (left panel) and CCRF-CEM (right panel). **b** Relative mRNA expression of MYC, SKP2, SIRT1, HES1 and p27 in SIRT1 knockdown MOLT-4 (top panel) and CCRF-CEM (bottom panel). **c-d** Western blotting for p27 following CHX treatment of SIRT1 knockdown or control MOLT-4 and CCRF-CEM (left panel). The right panel showed results of densitometry quantitation (n = 3). **e** Western blotting for p27 expression in SIRT1 WT and KO T-ALL. **f** Relative mRNA expression of SIRT1-exon4, p27, MYC, SKP2, HES1 in SIRT1 WT and KO T-ALL

To determine the potential target responsible for G1/S arrest induced by loss of SIRT1, multiple proteins associated with cell cycle entry, including MYC, SKP2, p16, p21, p27, cyclin E1, CDK4, CDK6, AKT and p-AKT were evaluated. The protein levels of p27 were markedly increased in ShSIRT1 transfected MOLT-4 and CCRF-CEM cells compared with control cells (Fig. 5a). Moreover, Q-PCR analysis demonstrated similar p27 mRNA levels in control and SIRT1 knockdown cells, suggesting that SIRT1 might decrease p27 protein levels but not the corresponding mRNA levels (Fig. 5b). As expected, the protein half-life of p27 was prolonged in SIRT1-specific ShRNA-transfected MOLT-4 and CCRF-CEM cells (Fig. 5c-d). Conversely, increased SIRT1 protein expression but not SIRT1-H363Y mutant reduced p27 protein levels (Fig. S5a-b). Western blot analysis with anti-SIRT1 (D1D7) rabbit monoclonal antibody (CST, #9475) and anti-SIRT1 (07–131) rabbit polyclonal antibody (Sigma-Aldrich, #07–131) revealed that SIRT1 KO T-ALL cells expressed the expected SIRT1 mutant protein with the in-frame deletion of exon 4 and that SIRT1 deficiency upregulated p27 protein expression but had little effect on p27 mRNA levels (Fig. 5e-f).

### SIRT1 regulates cell proliferation by repressing p27

**Fig. 6:**
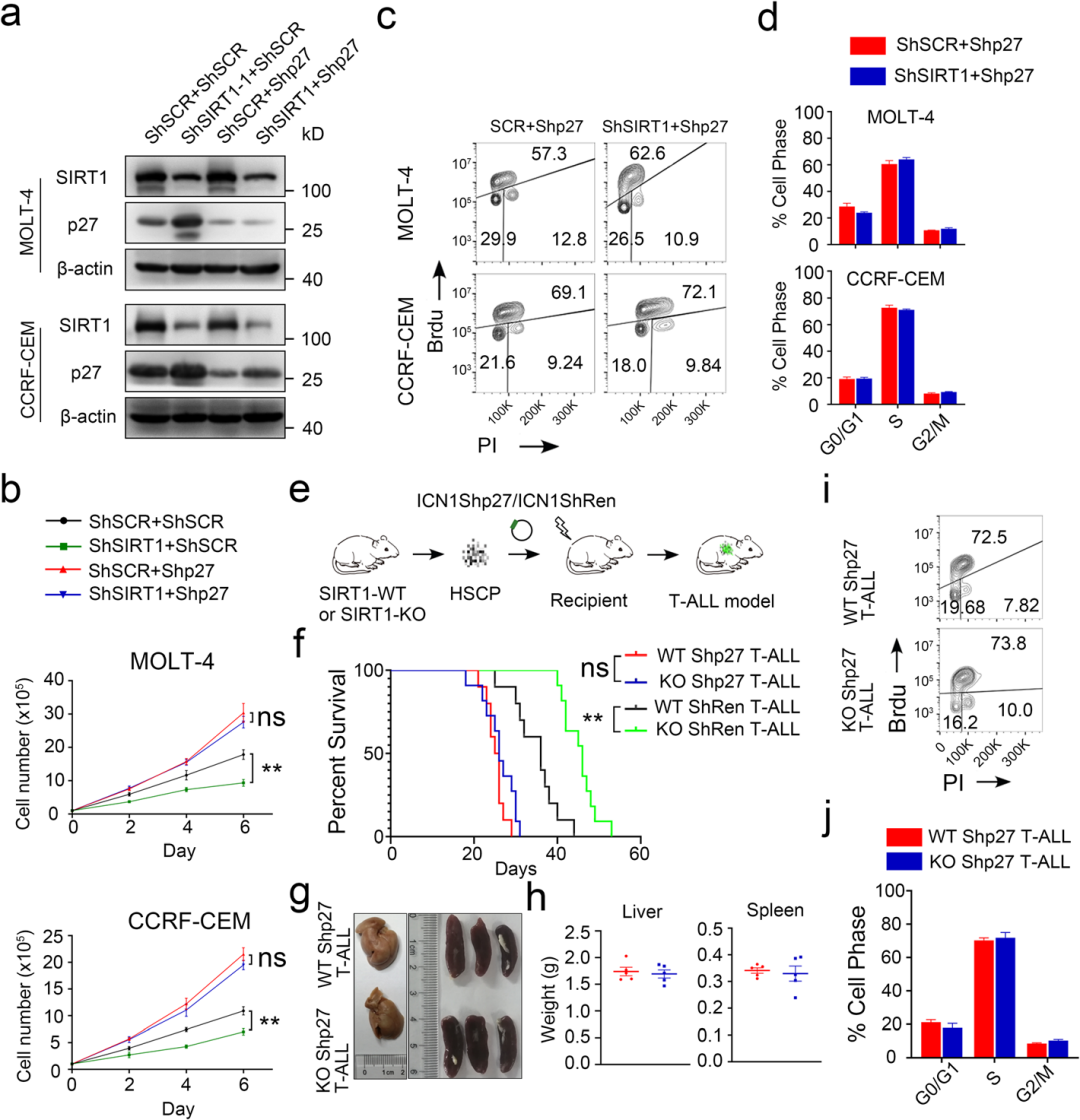
Inhibition of SIRT1 reduces T-ALL cell growth by activating p27. **a** SIRT1 and p27 protein levels were analyzed in MOLT-4 or CCRF-CEM cells co-transduced with ShSIRT1 and Shp27. **b** MOLT-4 (top panel) and CCRF-CEM (bottom panel) co-transduced with ShSIRT1 and Shp27 were counted at the indicated time points(n = 3). **c-d** Cell cycle phase distribution of MOLT-4 or CCRF-CEM cells co-transduced with ShSIRT1 and Shp27 were analyzed by FACS and quantified (n = 3). **e** Schematic representation of murine T-ALL model co-expressing Shp27. **f** Survival was compared among the recipient mice in **e**. **g-h** Representative images and weight quantification of spleens and livers. **i-j** Cell cycle phase distribution of WT and KO T-ALL expressing Shp27 were analyzed by FACS and quantified (n = 5 per group)

To confirm the importance of p27 in the inhibitory effects of ShSIRT1, Shp27 was used to knock down p27 expression in T-ALL cell lines expressing ShSIRT1–1 (Fig. 6a). The inhibitory effects of ShSIRT1 on proliferation were significantly reduced following p27 knockdown in MOLT-4 and CCRF-CEM cells (Fig. 6b). ShRNA-mediated knockdown of p27 significantly reduced SIRT1 knockdown-induced cell cycle arrest, as determined by BrdU-PI FACS analysis (Fig. 6c-d). We next evaluated the role of p27 in the SIRT1 KO T-ALL mouse model. A retroviral vector co-expressing Shp27 and ICN1 in one construct was used to transduce SIRT1 KO HSPCs (Fig. 5e). The efficiency of p27 knockdown was confirmed by western blot analysis (Fig. S6a). Moreover, SIRT1 KO did not decrease the peripheral blood or bone marrow leukemic burden in mice engrafted with p27-specific ShRNA-expressing T-ALL cells (Fig. 5f-h and S6b). Loss of SIRT1 did not induce cell cycle arrest in shp27 expressing T-ALL mouse model (Fig. 5i-j).

### SIRT1 promotes the ubiquitination and Thr187 phosphorylation of p27

**Fig. 7:**
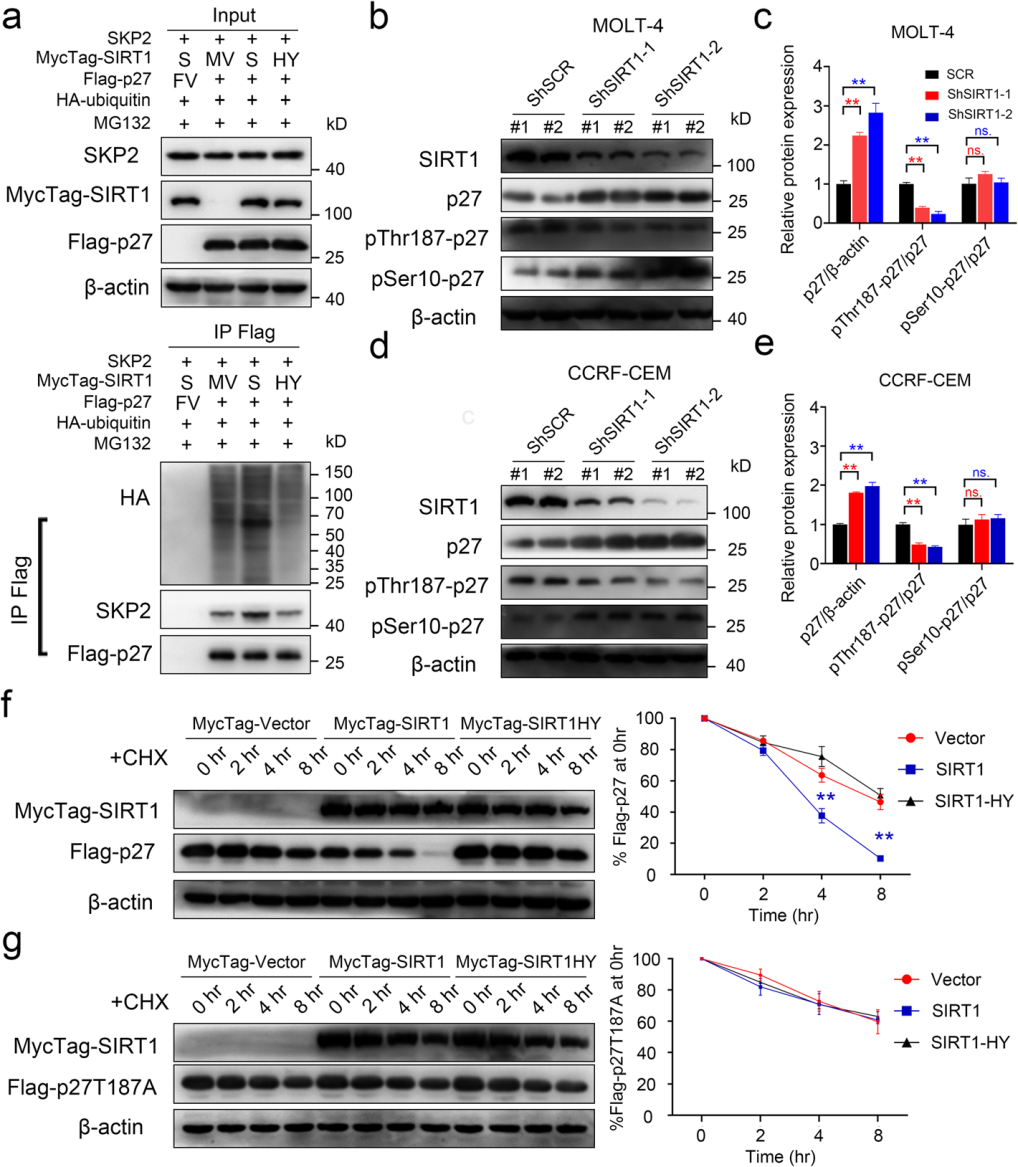
SIRT1 promotes ubiquitination and Thr-187 phosphorylation of p27. **a** 293 T cells were co-transfected with pCMV-N-Flag (FV), Flag-p27, pCMV-SKP2, pCMV-N-MycTag (MV), MycTag-SIRT1 (S), MycTag-SIRT1-H363Y (HY) and HA-tagged ubiquitin as indicated and subjected to ubiquitination analysis. **b-e** Western blot relative quantification analysis of Phospho-p27 and p27 levels in ShSIRT1 MOLT-4 and CCRF-CEM cells. **f-g** Western blotting for Flag-p27 or Flag-p27T187A following CHX treatment in cells from Fig. S7c or d (left panel). The right panel showed results of densitometry quantitation (n = 3)

The Notch target SKP2 is a major ubiquitin ligase that controls the abundance of cell cycle regulatory proteins, such as p21, p27 and p57 [11]. The interaction between p27 and SKP2 was confirmed by co-immunoprecipitation experiments in CCRF-CEM cells (Fig. S7a-b). As shown in Fig. 7a, overexpression of SIRT1 increased the ubiquitination of p27 and the interaction between p27 and SKP2 compared to treatment with the empty vector or SIRT1HY vector. It has been reported that the degradation of p27 is promoted by its phosphorylation [31, 38]. We assessed the phosphorylation of p27 in MOLT-4 and CCRF-CEM cells and found that silencing SIRT1 with ShRNA decreased the phosphorylation of p27-Thr187 but did not affect p27-S10 (Fig. 7b-e, Fig. S7c-d). Expression of wild-type SIRT1 decreased Flag-p27 protein expression and increased the phosphorylation of p27-Thr187 but did not affect p27-Ser10 (Fig. S7c). Expression of wild-type SIRT1 did not affect the p27T187A mutant protein levels (Fig. S7e). However, SIRT1H363Y did not affect the p27 and p27T187A mutant protein levels (Fig. S7f). CHX experiments indicated that SIRT1 accelerated the Flag-p27 protein degradation but did not affect the p27T187A mutant protein degradation (Fig. 7f, g).

### SIRT1 co-immunoprecipitates with CDK2 and induces CDK2 deacetylation

**Fig. 8:**
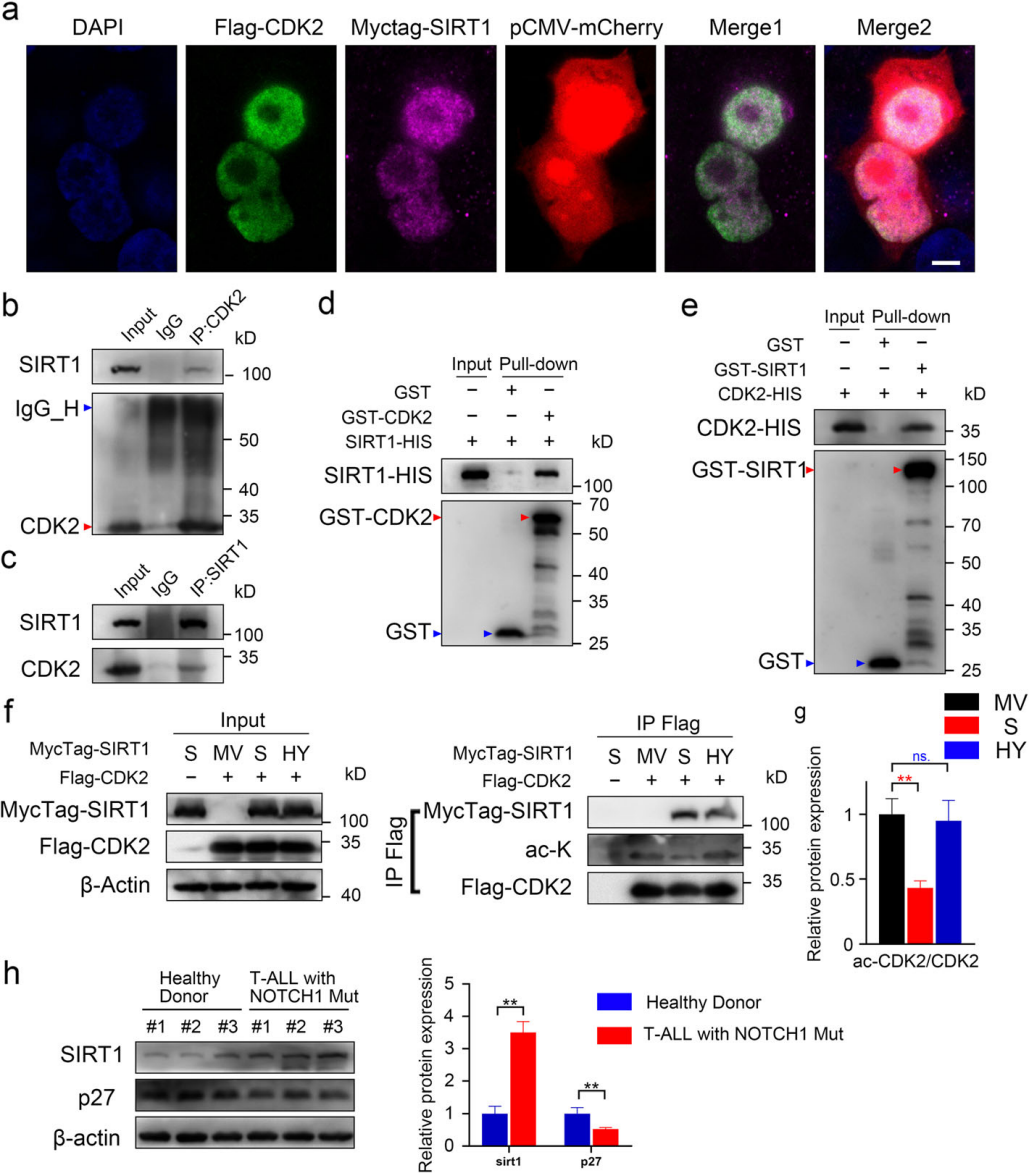
SIRT1 co-immunoprecipitates with CDK2 and induces CDK2 deacetylation. a 293 T cells were transfected with pCMV-mCherry, Flag-CDK2, MycTag-SIRT1 for cellular localization analysis (scale = 5 μm). Cells were immunostained with DAPI (blue) for nucleus, CDK2 (green) and SIRT1 (purple). **b-c** Endogenous immunoprecipitation of SIRT1 and CDK2 was performed in CCRF-CEM cells. **d-e** Purified GST-tagged SIRT1, CDK2 and HIS-tagged SIRT1, CDK2 were used for in vitro binding assay. **f-g** Immunoprecipitation was performed using anti-Flag magnetic beads on lysates derived from 293 T cells transfected as indicated. Western blot relative quantification analysis of acetylated CDK2 detected by anti-acetylated-lysine antibody (ac-K). **h** Immunoblots of SIRT1 and p27 in blood cells from 3 healthy donor and 3 primary T-ALL patient samples with NOTCH1 mutation

A previous study proved that CDK2 can directly phosphorylate p27 at Thr187 [38]. We co-transfected 293 T cells with a Flag-CDK2 vector, a Myctag-SIRT1 vector and a pCMV-mCherry vector. Immunofluorescence showed that both SIRT1 and CDK2 mainly co-localized in the nucleus (Fig. 8a). Co-transfection of vectors expressing Myctag-SIRT1 and Flag-CDK2 in 293 T cells revealed that SIRT1 directly interacts with CDK2 (Fig. S7g-h). Endogenous protein interaction between SIRT1 and CDK2 is detected in CCRF-CEM cells (Fig. 8b-c). In vitro GST pulldown assays also showed interaction between SIRT1 and CDK2 (Fig. 8d-e). Acetylation has been reported to downregulate CDK2-mediated phosphorylation, while deacetylation upregulates it [17, 28]. We found that SIRT1 but not the SIRT1-H363Y mutant decreased the acetylation level of CDK2 (Fig. 8f-g). The ubiquitination assay showed that overexpression of CDK2 increased the polyubiquitination of p27 (Fig. S7i). However, the polyubiquitination of Flag-p27 was only slightly increased in 293 T cells transfected with SIRT1 (Fig. S7j). This may be due to a relatively low endogenous expression of SKP2 in 293 T cells. We performed a western blot assay in blood cells from 3 healthy donor and 3 primary T-ALL patient samples. The result revealed that primary T-ALL cells harboring NOTCH1 mutations showed higher SIRT1 protein expression and lower p27 protein expression than normal blood cells from healthy donor (Fig. 8h).

## Discussion

T-ALL is an aggressive hematological malignancy characterized by the diffuse infiltration of the bone marrow by immature T cells. T-ALL results from a multistep process of genetic alterations in critical genes which regulate cell proliferation, growth, survival and differentiation during thymocyte development.

In the present study, we demonstrated that SIRT1 is upregulated in T-ALL and could promote proliferation of T-ALL cells and plays an important role in promoting leukemia development in T-ALL by targeting p27 for ubiquitin-mediated proteasomal degradation. Our study revealed an important role for SIRT1 and p27 in regulating cell cycle progression in T-ALL.

The deregulation of cell cycle control is a hallmark of cancer and plays a critical role in the molecular pathogenesis of T-ALL. Silencing of the tumor suppressor p16INK4a and p14ARF by chromosomal deletions occurs frequently in T-ALL [10, 12]. 15% of T-ALL patients are known to have chromosomal deletions in 13q14.2 resulting in the loss of retinoblastoma 1(RB1), which encodes a global cell cycle regulator [10]. Moreover, approximately 12% of T-ALL patients have genetic deletions around 12p13.2 involving the CDKN1B gene, which encodes the tumor suppressor p27kip1 [36]. In addition, oncogenic Notch1 signaling has been shown to promote cell proliferation by impacting G1/S cell cycle progression. In T-ALL, Notch1 activation upregulates the cell cycle genes CCND3, CDK4, CDK6 and CCND1 and induces CDK2 activity [18, 37]. Consistently, inhibition of Notch signaling in T-ALL cell lines triggers upregulation of the cyclin-dependent kinase inhibitors CDKN2D (p19/INK4d) and CDKN1B (p27/Kip1) [35]. Moreover, Notch1 can further promote the transcription of S phase kinase-associated protein 2 (SKP2), which mediates the proteasomal degradation of the cell cycle inhibitors CDKN1B (p27/Kip1) and CDKN1A (p21/Cip1) [9].

Previous studies have implicated SIRT1 in the control of cell cycle progression. Knockdown of SIRT1 in HeLa cells upregulates of p16 transcriptional and protein levels [34]. Moreover, silencing SIRT1 in MSCs significantly enhances the expression of p16 and p21 at the protein level [7]. SIRT1 inhibitors increase the extent of G1 arrest through the SIRT1/P53/P21 axis [3]. SIRT1 silencing dramatically suppresses the proliferation of non-small cell lung cancer (NSCLC) cells by decreasing p27/Kip1 protein stability [45]. In our study, we assessed the protein levels of key proteins involved in the cell cycle, including those involved in CDK2, CDK4, CDK6, p21, p16 and p27 signaling pathways. Only the p27 protein level was significantly changed following SIRT1 knockdown in T-ALL.

The expression of p27 is controlled by transcriptional, translational and post-translational mechanisms. FoxO transcription factors have been shown to bind to the promoter of p27 and transcriptionally activate p27 [29]. In addition, F-box protein SKP2 specifically recognizes p27 for ubiquitination and degradation [4]. PI3K signaling has also been shown to be involved in reducing the levels of p27 by 2 mechanisms. First, PI3K mediates activation of the kinase AKT, which phosphorylates and deactivates Forkhead/FoxO transcription factors, leading to reduced p27 transcription [20]. Second, PI3K induces transcription of SKP2 which binds to p27 to promote proteasomal degradation [2]. In our study, no significant changes in the protein levels of SKP2 or phosphorylation levels of AKT were observed between ShSIRT1 and ShSCR T-ALL cells. However, we found that overexpression of SIRT1 increased the ubiquitination of p27 and the interaction between p27 and SKP2.

There is now growing recognition of the essential roles of lysine acetylation in protein degradation mediated by the E3 ligase SKP2. It was reported that SKP2 directly interacts with p300 and negatively affects the ability of P300 to modulate the functions of p53 in apoptosis [19]. Furthermore, P300 has been shown to affect SKP2 function in an acetylation-dependent manner and SIRT3 was reported to interact with SKP2 and regulate the SKP2 acetylation status [16]. It was also reported that SIRT2 deacetylates SKP2 and induces subsequent degradation [26]. Another study provided an additional mechanism by which deacetylation of FOXO3 by SIRT1 or SIRT2 facilitates SKP2 docking on FOXO3 for proteasomal degradation [41]. As shown in Fig. S8, we found that SIRT1 decreased the acetylation level of CDK2 and increased the phosphorylation of p27 at Thr187. pThr187-p27 accelerates degradation mediated by the E3 ligase SKP2 during the G1/S phase transition [14].

Our study had some limitations. First, we did not detect the p27 acetylation level. Whether SIRT1 regulates p27 degradation by regulating its acetylation level needs more evidence. Second, we did not compare downstream gene expression after silencing SIRT1 in DND-41 and KOPT-K1 T-ALL cell lines to determine the factors that lead to the opposite observations reported by Okasha S M et al. [33] .

## Conclusion

In summary, our study shows that SIRT1 is upregulated by MYC in Notch-induced T-ALL and involved in the cell cycle of T-ALL cells by deacetylating CDK2 and promotes the phosphorylation and subsequent degradation of p27. Our findings offer a new molecular mechanism by which SIRT1 regulates cell cycle of T-ALL cells by promoting p27 degradation and suggest that SIRT1 is a promising target in T-ALL.

## Supplementary Information

**Additional file 1: Supplementary Fig. 1**. Notch-induced T-ALL model. a Peripheral blood (PB) percentage and CD4^+^ CD8^+^ immunophenotype of GFP^+^ cells were analyzed by FACS at 4 weeks after transplantation. b Representative images of the sizes and quantification of the weight of spleens of recipient mice. c Histological analysis of parenchymal organs from spleen, liver, and bone performed at 4 weeks after transplantation (scale = 5 mm).

**Additional file 2: Supplementary Fig. 2**. Increased SIRT1 protein levels in T-ALL with Notch1 mutation. a-b Distributions of SIRT1 mRNA expression derived from T-ALL cells treated with GSI and washed out. c-d Relative mRNA expression of SIRT1 in MYC knockdown MOLT-4 and CCRF-CEM cells. e-f Representative protein expression of SIRT1 in T-ALL cells treated with GSI after infection with plasmids encoding MYC or vector.

**Additional file 3: Supplementary Fig. 3**. SIRT1 knockdown impairs proliferation. a Sanger sequencing result of plasmid encoding SIRT1-H363Y mutant. b-c T-ALL cells were treated with increasing concentrations of nicotinamide for 24 h, and cell viability was measured by CCK-8 assays.

**Additional file 4: Supplementary Fig. 4**. Effects of SIRT1 loss on Notch-induced leukemia. a Schematic representation of Mouse SIRT1 wild-type allele (WT), conditional targeted allele (CO), and knockout allele (KO). b Schematic representation of ablation of Sirt1 induced by poly I:C treatment. c Genotyping analysis of tail DNA from SIRT1^+/+^ (550 bp), SIRT1^CO/+^ (550 bp and 742 bp) and SIRT1^CO/CO^ (742 bp). d Genotyping analysis of cDNA from SIRT1^+/+^ (489 bp), SIRT1^−/+^ (489 bp and 336 bp) and SIRT1^−/−^ (336 bp). e Peripheral blood GFP^+^ cells from SIRT1 KO and WT T-ALL were analyzed by FACS for CD4^+^ CD8^+^ immunophenotype. f Analysis of homing ability of SIRT1 KO and WT BM cells (*n* = 5 for each group). Mean values (± SEM) are shown. g Analysis of homing ability of SIRT1 KO and WT T-ALL cells (n = 5 for each group). Mean values (± SEM) are shown.

**Additional file 5: Supplementary Fig. 5**. SIRT1 decreases p27 protein levels. a-b SIRT1 and p27 protein levels were analyzed in MOLT-4 or CCRF-CEM cells from Fig. 3f.

**Additional file 6: Supplementary Fig. 6**. SIRT1 regulates T-ALL development by p27. a Western blotting for SIRT1 and p27 expression in SIRT1 WT and KO Shp27 T-ALL. b Histological analysis of spleens and livers (scale = 5 mm).

**Additional file 7: Supplementary Fig. 7**. SIRT1 co-immunoprecipitates with CDK2 and promotes the ubiquitination and Thr187 phosphorylation of p27. a-b Endogenous immunoprecipitation of SKP2 and p27 was performed in CCRF-CEM cells. IgG_H: IgG heavy chain. Ma-p27: mouse p27 antibody. Ra-p27: Rabbit p27 antibody. c-d Western blot relative quantification analysis of Phospho-p27 and p27 levels in ShSIRT1 MOLT-4 and CCRF-CEM cells. e Phospho-p27, Flag-p27 levels were detected in 293 T cells transfected with MycTag-SIRT1 and Flag-p27 as indicated. f Phospho-p27, Flag-p27T187A levels were detected in 293 T cells transfected with MycTag-SIRT1 and Flag-p27T187A as indicated. g-h Immunoprecipitation was performed using anti-Flag or anti-Myc magnetic beads on lysates derived from 293 T cells expressing Flag-CDK2 and MycTag-SIRT1. i 293 T cells were co-transfected with Flag-p27, pCMV-N-Flag (FV), pCMV-Blank (V), pCMV-CDK2 and HA-tagged ubiquitin as indicated and subjected to ubiquitination analysis. j 293 T cells were co-transfected with Flag-p27, pCMV-N-Flag (FV), pCMV-Blank (V), MycTag-SIRT1, MycTag-SIRT1-H363Y and HA-tagged ubiquitin as indicated and subjected to ubiquitination analysis.

**Additional file 8: Supplementary Fig. 8**. Schematic representation of SIRT1 regulating p27 in T-ALL.

**Additional file 9: Supplementary Table 1**. Human patient samples.

**Additional file 10: Supplementary Table 2**. The antibodies used in this study.

**Additional file 11: Supplementary Table 3**. Primers and oligonucleotides.

## Data Availability

The datasets used and analyzed during the current study are available from the corresponding author on reasonable request.

## Abbreviations

T-ALL: Acute T-cell lymphoblastic leukemia
GSI: γ-secretase inhibitor
HSPC: Hematopoietic stem/progenitor cells
ICN1: Intracellular Notch1
CDK2: Cyclin-dependent kinases 2
SKP2: S-phase kinase-associated protein 2
