# Supplementary figures and images for: SIRT1 regulates the phosphorylation and degradation of P27 by deacetylating CDK2 to promote T-cell acute lymphoblastic leukemia progression
Source: J Exp Clin Cancer Res. 2021 Aug 18;40:259. doi: 10.1186/s13046-021-02071-w (PMC8371879; doi:10.1186/s13046-021-02071-w)

a

PB 4 weeks

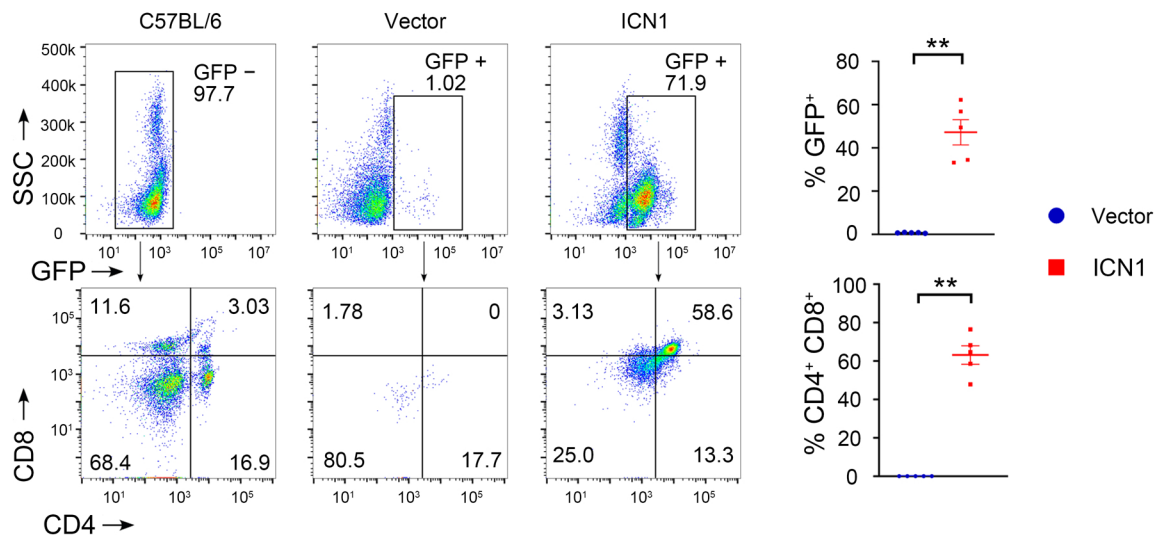

b

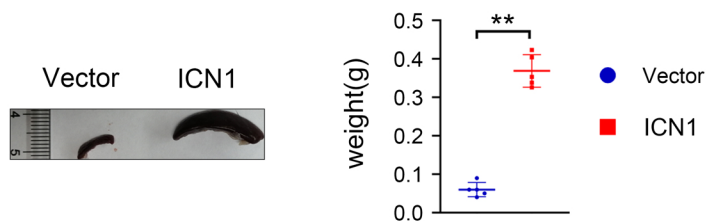

c

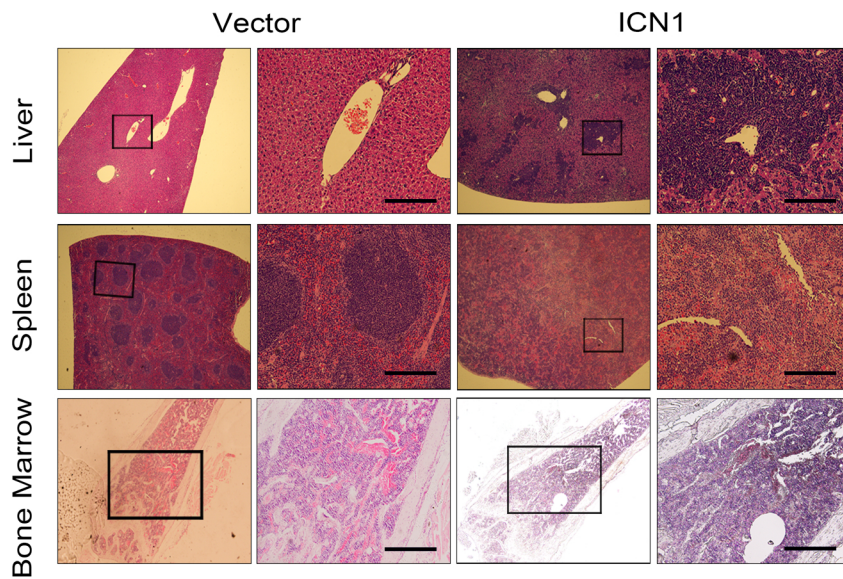

Supplement: Supplementary file 1 — Additional file 1: Supplementary Fig. 1. Notch-induced T-ALL model. a Peripheral blood (PB) percentage and CD4+ CD8+ immunophenotype of GFP+ cells were analyzed by FACS at 4 weeks after transplantation. b Representative images of the sizes and quantification of the weight of spleens of recipient mice. c Histological analysis of parenchymal organs from spleen, liver, and bone performed at 4 weeks after transplantation (scale = 5 mm). [file 13046_2021_2071_MOESM1_ESM.pdf]

**a**

DMSO GSI Mock Wash

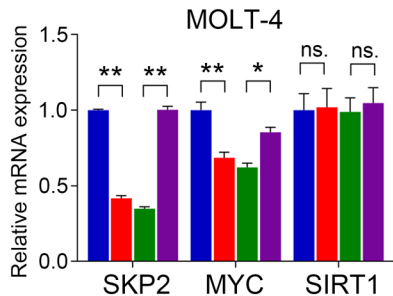**b**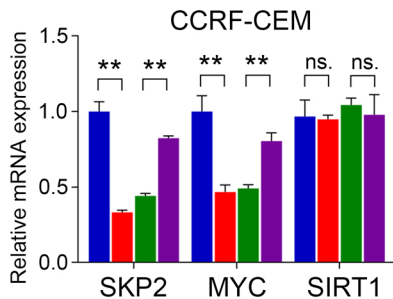**c**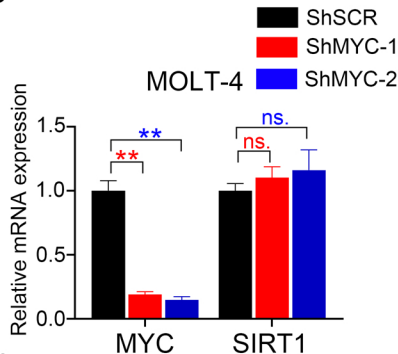**d**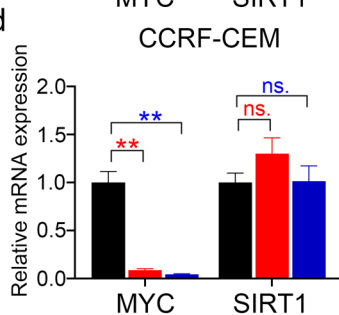**e**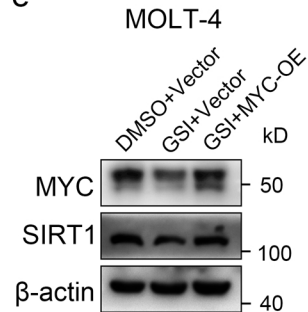**f**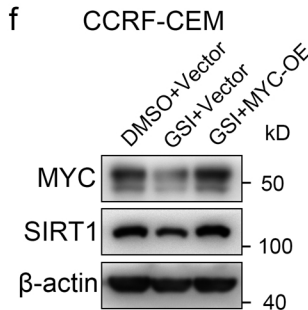

Supplement: Supplementary file 2 — Additional file 2: Supplementary Fig. 2. Increased SIRT1 protein levels in T-ALL with Notch1 mutation. a-b Distributions of SIRT1 mRNA expression derived from T-ALL cells treated with GSI and washed out. c-d Relative mRNA expression of SIRT1 in MYC knockdown MOLT-4 and CCRF-CEM cells. e-f Representative protein expression of SIRT1 in T-ALL cells treated with GSI after infection with plasmids encoding MYC or vector. [file 13046_2021_2071_MOESM2_ESM.pdf]

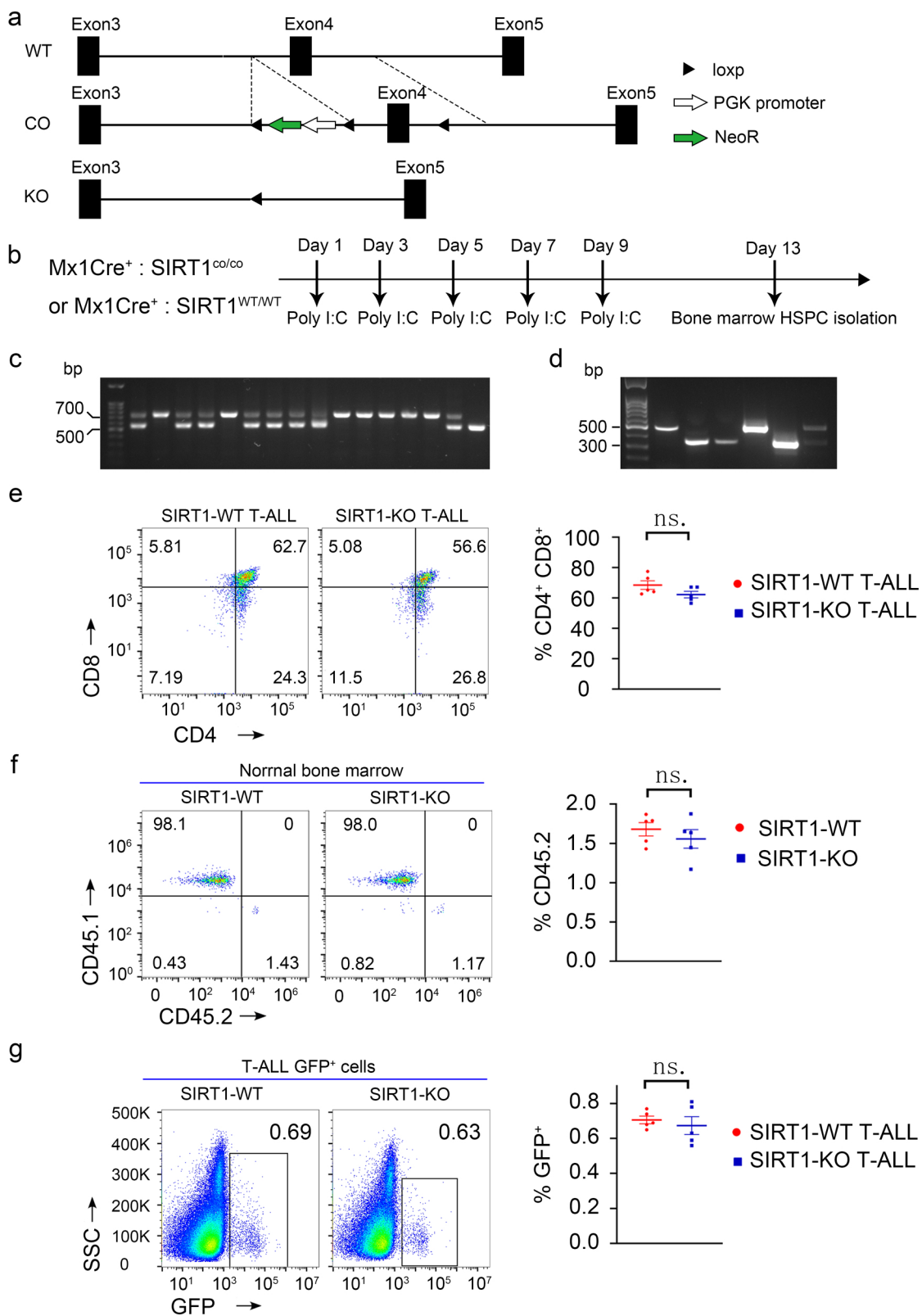

Supplement: Supplementary file 4 — Additional file 4: Supplementary Fig. 4. Effects of SIRT1 loss on Notch-induced leukemia. a Schematic representation of Mouse SIRT1 wild-type allele (WT), conditional targeted allele (CO), and knockout allele (KO). b Schematic representation of ablation of Sirt1 induced by poly I:C treatment. c Genotyping analysis of tail DNA from SIRT1+/+ (550 bp), SIRT1CO/+ (550 bp and 742 bp) and SIRT1CO/CO (742 bp). d Genotyping analysis of cDNA from SIRT1+/+ (489 bp), SIRT1−/+ (489 bp and 336 bp) and SIRT1−/− (336 bp). e Peripheral blood GFP+ cells from SIRT1 KO and WT T-ALL were analyzed by FACS for CD4+ CD8+ immunophenotype. f Analysis of homing ability of SIRT1 KO and WT BM cells (n = 5 for each group). Mean values (± SEM) are shown. g Analysis of homing ability of SIRT1 KO and WT T-ALL cells (n = 5 for each group). Mean values (± SEM) are shown. [file 13046_2021_2071_MOESM4_ESM.pdf]

a

## MOLT-4

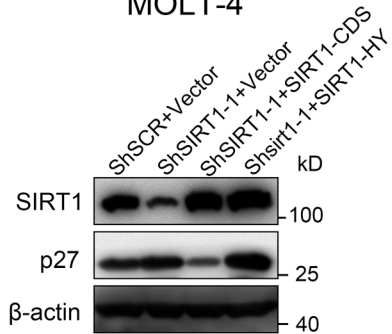

b

## CCRF-CEM

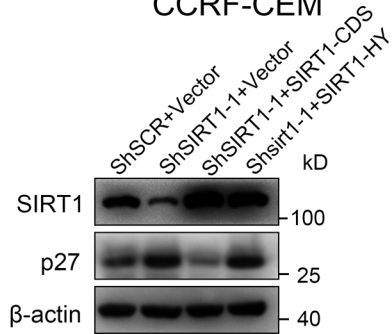

-

Supplement: Supplementary file 5 — Additional file 5: Supplementary Fig. 5. SIRT1 decreases p27 protein levels. a-b SIRT1 and p27 protein levels were analyzed in MOLT-4 or CCRF-CEM cells from Fig. 3f. [file 13046_2021_2071_MOESM5_ESM.pdf]

a

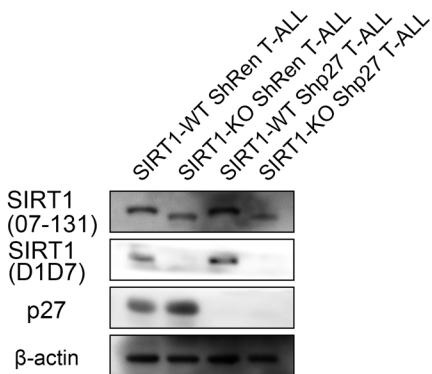

b

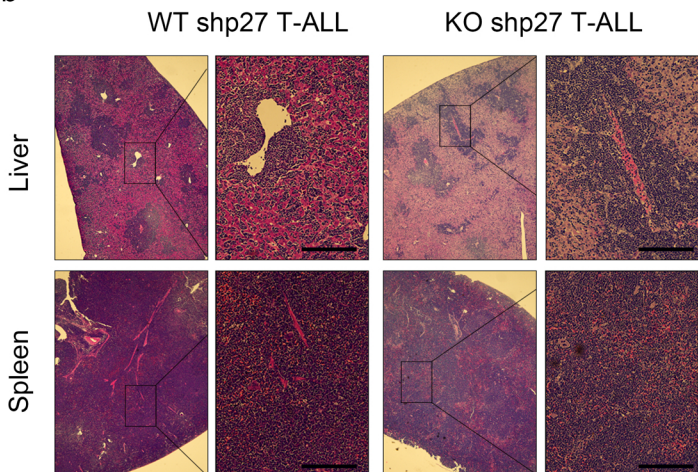

Supplement: Supplementary file 6 — Additional file 6: Supplementary Fig. 6. SIRT1 regulates T-ALL development by p27. a Western blotting for SIRT1 and p27 expression in SIRT1 WT and KO Shp27 T-ALL. b Histological analysis of spleens and livers (scale = 5 mm). [file 13046_2021_2071_MOESM6_ESM.pdf]

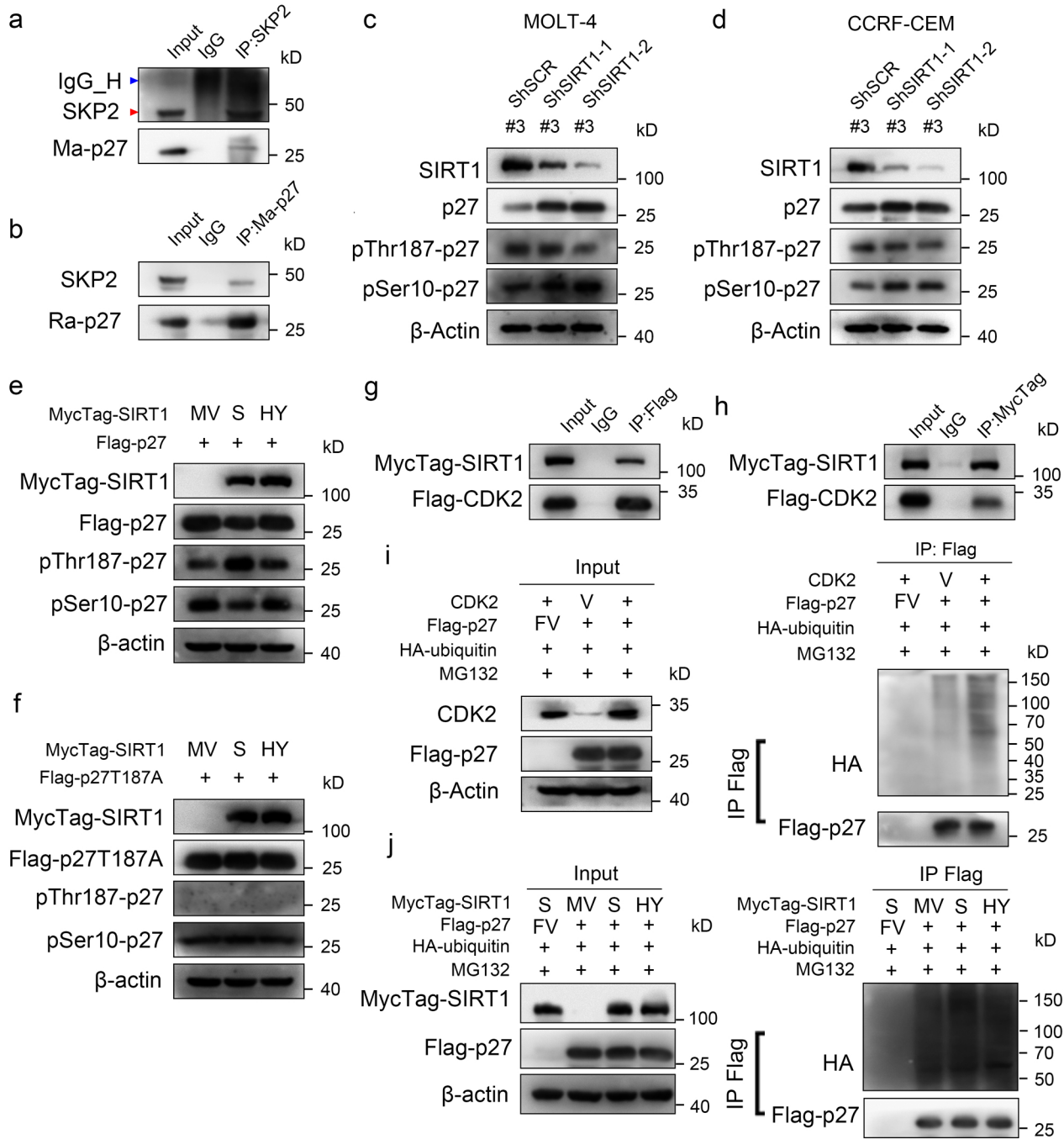

Supplement: Supplementary file 7 — Additional file 7: Supplementary Fig. 7. SIRT1 co-immunoprecipitates with CDK2 and promotes the ubiquitination and Thr187 phosphorylation of p27. a-b Endogenous immunoprecipitation of SKP2 and p27 was performed in CCRF-CEM cells. IgG_H: IgG heavy chain. Ma-p27: mouse p27 antibody. Ra-p27: Rabbit p27 antibody. c-d Western blot relative quantification analysis of Phospho-p27 and p27 levels in ShSIRT1 MOLT-4 and CCRF-CEM cells. e Phospho-p27, Flag-p27 levels were detected in 293 T cells transfected with MycTag-SIRT1 and Flag-p27 as indicated. f Phospho-p27, Flag-p27T187A levels were detected in 293 T cells transfected with MycTag-SIRT1 and Flag-p27T187A as indicated. g-h Immunoprecipitation was performed using anti-Flag or anti-Myc magnetic beads on lysates derived from 293 T cells expressing Flag-CDK2 and MycTag-SIRT1. i 293 T cells were co-transfected with Flag-p27, pCMV-N-Flag (FV), pCMV-Blank (V), pCMV-CDK2 and HA-tagged ubiquitin as indicated and subjected to ubiquitination analysis. j 293 T cells were co-transfected with Flag-p27, pCMV-N-Flag (FV), pCMV-Blank (V), MycTag-SIRT1, MycTag-SIRT1-H363Y and HA-tagged ubiquitin as indicated and subjected to ubiquitination analysis. [file 13046_2021_2071_MOESM7_ESM.pdf]

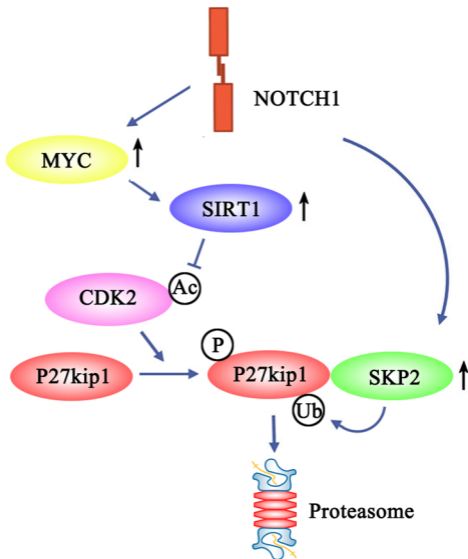

Supplement: Supplementary file 8 — Additional file 8: Supplementary Fig. 8. Schematic representation of SIRT1 regulating p27 in T-ALL. [file 13046_2021_2071_MOESM8_ESM.pdf]
