# Supplementary material for: SIRT1 regulates the phosphorylation and degradation of P27 by deacetylating CDK2 to promote T-cell acute lymphoblastic leukemia progression
Source: J Exp Clin Cancer Res. 2021 Aug 18;40:259. doi: 10.1186/s13046-021-02071-w (PMC8371879; doi:10.1186/s13046-021-02071-w)
Supplement: Supplementary file 3 — Additional file 3: Supplementary Fig. 3. SIRT1 knockdown impairs proliferation. a Sanger sequencing result of plasmid encoding SIRT1-H363Y mutant. b-c T-ALL cells were treated with increasing concentrations of nicotinamide for 24 h, and cell viability was measured by CCK-8 assays. [file 13046_2021_2071_MOESM3_ESM.pdf]

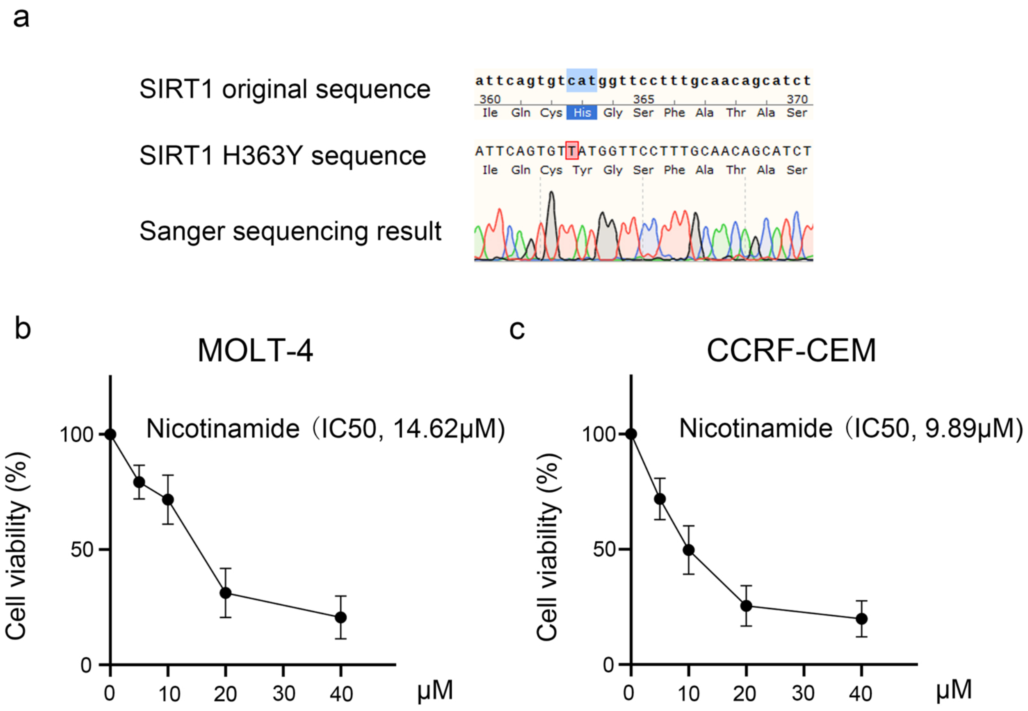

a
SIRT1 original sequence
attcagtgtcatggttcctttgcaacagcatct
360
365
370
Ile Gln Cys His Gly Ser Phe Ala Thr Ala Ser
SIRT1 H363Y sequence
ATTCAGTGTTATGGTTCCTTTGCAACAGCATCT
Ile Gln Cys Tyr Gly Ser Phe Ala Thr Ala Ser
Sanger sequencing result
b
MOLT-4
Nicotinamide（IC50, 14.62μM)
Cell viability (%)
100
50
0
0
10
20
30
40
μM
c
CCRF-CEM
Nicotinamide（IC50, 9.89μM)
Cell viability (%)
100
50
0
0
10
20
30
40
μM
