# Supplementary material for: SIRT1 regulates the phosphorylation and degradation of P27 by deacetylating CDK2 to promote T-cell acute lymphoblastic leukemia progression
Source: J Exp Clin Cancer Res. 2021 Aug 18;40:259. doi: 10.1186/s13046-021-02071-w (PMC8371879; doi:10.1186/s13046-021-02071-w)
Supplement: Supplementary file 9 — Additional file 9: Supplementary Table 1. Human patient samples. [file 13046_2021_2071_MOESM9_ESM.docx]

| **Table 1. Human patient samples** | | | | | |
| --- | --- | --- | --- | --- | --- |
|  | **Gender** | **Age** | **Source** | **Disease** | **NOTCH1 mutation** |
| Patient 1# | Female | 57 | BM | T-ALL | HD mutation |
| Patient 2# | Male | 41 | BM | T-ALL | HD mutation |
| Patient 3# | Female | 35 | BM | T-ALL | HD+PEST mutation |
| Healthy Donor 1# | Male | 28 | PB | No | - |
| Healthy Donor 2# | Male | 44 | PB | No | - |
| Healthy Donor 3# | Female | 35 | PB | No | - |
