# Supplementary material for: SIRT1 regulates the phosphorylation and degradation of P27 by deacetylating CDK2 to promote T-cell acute lymphoblastic leukemia progression
Source: J Exp Clin Cancer Res. 2021 Aug 18;40:259. doi: 10.1186/s13046-021-02071-w (PMC8371879; doi:10.1186/s13046-021-02071-w)
Supplement: Supplementary file 10 — Additional file 10: Supplementary Table 2. The antibodies used in this study. [file 13046_2021_2071_MOESM10_ESM.docx]

| **Table 2. The antibodies used in this study.** | | |
| --- | --- | --- |
| **Antibodies for western blot** | **Suppliers** | **Catalogue numbers** |
| SIRT1 (D1D7) Rabbit mAb | Cell Signaling Technology | Cat no. #9475 |
| β-Actin (8H10D10) Mouse mAb | Cell Signaling Technology | Cat no. #3700 |
| FLAG tag (D6W5B) Rabbit mAb | Cell Signaling Technology | Cat no. #14793 |
| FLAG tag (9A3) Mouse mAb | Cell Signaling Technology | Cat no. #8146 |
| AKT Rabbit mAb | Cell Signaling Technology | Cat no. #9272 |
| Phospho-AKT (Ser473) Rabbit mAb | Cell Signaling Technology | Cat no. #4060 |
| Phospho-AKT (Thr308) Rabbit mAb | Cell Signaling Technology | Cat no. #13038 |
| CDK6 (D4S8S) Rabbit mAb | Cell Signaling Technology | Cat no. #13331 |
| Myc tag (9B11) Mouse mAb | Cell Signaling Technology | Cat no. # 2276 |
| CDK4 [EPR4513-32-7] Rabbit mAb | Abcam | Cat no. #ab108357 |
| Cyclin E1 [EP435E] Rabbit mAb | Abcam | Cat no. #ab33911 |
| P27 KIP 1[Y236] Rabbit mAb | Abcam | Cat no. #ab32034 |
| P21 [EPR362] Rabbit mAb | Abcam | Cat no. # ab109520 |
| P16 ARC [EP1551Y] Rabbit mAb | Abcam | Cat no. # ab51243 |
| HA tag (HA.C5) Mouse mAb | Abcam | Cat no. #ab18181 |
| c-Myc [Y69] Rabbit mAb | Abcam | Cat no. #ab32072 |
| SKP2 [EPR3305(2)] Rabbit mAb | Abcam | Cat no. # ab183039 |
| Anti-SIRT1 Rabbit antibody | Millipore | Cat no. # 07-131 |
| GAPDH Rabbit mAb | Bioss | Cat no. # bs-0755R |
