# Supplementary material for: SIRT1 regulates the phosphorylation and degradation of P27 by deacetylating CDK2 to promote T-cell acute lymphoblastic leukemia progression
Source: J Exp Clin Cancer Res. 2021 Aug 18;40:259. doi: 10.1186/s13046-021-02071-w (PMC8371879; doi:10.1186/s13046-021-02071-w)
Supplement: Supplementary file 11 — Additional file 11: Supplementary Table 3. Primers and oligonucleotides. [file 13046_2021_2071_MOESM11_ESM.docx]

| **Table 3. Primers and oligonucleotides** | | |
| --- | --- | --- |
| **Primers name** | **Sequence** | |
| Human SKP2(Q-PCR) | Forward | ATGCCCCAATCTTGTCCATCT |
|  | Reverse | CACCGACTGAGTGATAGGTGT |
| Human MYC(Q-PCR) | Forward | TCCCTCCACTCGGAAGGAC |
|  | Reverse | CTGGTGCATTTTCGGTTGTTG |
| Human SIRT1(Q-PCR) | Forward | TGTGTCATAGGTTAGGTGGTGA |
|  | Reverse | AGCCAATTCTTTTTGTGTTCGTG |
| Human GAPDH(Q-PCR) | Forward | CTGGGCTACACTGAGCACC |
|  | Reverse | AAGTGGTCGTTGAGGGCAATG |
| Mouse SKP2(Q-PCR) | Forward | ATGGACTGCTCTCAAACCTCG |
|  | Reverse | CCTGGAAAGTTCTCCCGACTAA |
| Mouse MYC(Q-PCR) | Forward | ATGCCCCTCAACGTGAACTTC |
|  | Reverse | CGCAACATAGGATGGAGAGCA |
| Mouse SIRT1(Q-PCR) | Forward | ATGACGCTGTGGCAGATTGTT |
|  | Reverse | CCGCAAGGCGAGCATAGAT |
| Mouse GAPDH(Q-PCR) | Forward | TGACCTCAACTACATGGTCTACA |
|  | Reverse | CTTCCCATTCTCGGCCTTG |
| Human NOTCH(Q-PCR) | Forward | GCCTTGCTGCCAGCGCCC |
|  | Reverse | CCAGTGGCTGCACGTCTGC |
| Human HES1 (Q-PCR) | Forward | TCAACACGACACCGGATAAAC |
|  | Reverse | GCCGCGAGCTATCTTTCTTCA |
| Human CDKN1B (Q-PCR) | Forward | TAATTGGGGCTCCGGCTAACT |
|  | Reverse | TGCAGGTCGCTTCCTTATTCC |
| Mouse CDKN1B (Q-PCR) | Forward | TCAAACGTGAGAGTGTCTAACG |
|  | Reverse | CCGGGCCGAAGAGATTTCTG |
| Mouse HES1 (Q-PCR) | Forward | CCAGCCAGTGTCAACACGA |
|  | Reverse | AATGCCGGGAGCTATCTTTCT |
| Mouse SIRT1 exon4(Q-PCR) | Forward | TCTCCTGTGGGATTCCTGACT |
|  | Reverse | ACTTGAAGAATGGTCTTGGGTCT |
| Mouse SIRT1-co-gDNA | Forward | GGTTGACTTAGGTCTTGTCTG |
|  | Reverse | CGTCCCTTGTAATGTTTCCC |
| Mouse SIRT1-ko-cDNA | Forward | GGCACCGATCCTCGAACAAT |
|  | Reverse | TTGGATTCCTGCAACCTGCT |
